# Hepatitis C virus alters the morphology and function of peroxisomes

**DOI:** 10.3389/fmicb.2023.1254728

**Published:** 2023-09-21

**Authors:** Esther Martin de Fourchambault, Nathalie Callens, Jean-Michel Saliou, Marie Fourcot, Oceane Delos, Nicolas Barois, Quentin Thorel, Santseharay Ramirez, Jens Bukh, Laurence Cocquerel, Justine Bertrand-Michel, Guillemette Marot, Yasmine Sebti, Jean Dubuisson, Yves Rouillé

**Affiliations:** ^1^Université de Lille, CNRS, Inserm, CHU Lille, Institut Pasteur de Lille, U 1019 – UMR9017 – CIIL – Center for Infection and Immunity of Lille, Lille, France; ^2^Université de Lille, CNRS, Inserm, CHU Lille, Institut Pasteur de Lille, UAR CNRS 2014 - US Inserm 41 - PLBS, Lille, France; ^3^MetaToul-MetaboHUB, National Infrastructure of Metabolomics and Fluxomics, Toulouse, France; ^4^I2MC, Université de Toulouse, Inserm, Université Toulouse III – Paul Sabatier (UPS), Toulouse, France; ^5^Université de Lille, Inserm, CHU Lille, Institut Pasteur de Lille, U1011 - EGID, Lille, France; ^6^Faculty of Health and Medical Sciences, Copenhagen Hepatitis C Program (CO-HEP), Department of Infectious Diseases, Copenhagen University Hospital Hvidovre and Department of Immunology and Microbiology, University of Copenhagen, Copenhagen, Denmark; ^7^Université de Lille, Inria, CHU Lille, ULR 2694 - METRICS: Évaluation des technologies de santé et des pratiques médicales, Lille, France

**Keywords:** hepatitis C virus, HCV genotype, peroxisome, proximity biotinylation, APEX2, CRISPR-Cas9, ROS

## Abstract

Despite the introduction of effective treatments for hepatitis C in clinics, issues remain regarding the liver disease induced by chronic hepatitis C virus (HCV) infection. HCV is known to disturb the metabolism of infected cells, especially lipid metabolism and redox balance, but the mechanisms leading to HCV-induced pathogenesis are still poorly understood. In an APEX2-based proximity biotinylation screen, we identified ACBD5, a peroxisome membrane protein, as located in the vicinity of HCV replication complexes. Confocal microscopy confirmed the relocation of peroxisomes near HCV replication complexes and indicated that their morphology and number are altered in approximately 30% of infected Huh-7 cells. Peroxisomes are small versatile organelles involved among other functions in lipid metabolism and ROS regulation. To determine their importance in the HCV life cycle, we generated Huh-7 cells devoid of peroxisomes by inactivating the PEX5 and PEX3 genes using CRISPR/Cas9 and found that the absence of peroxisomes had no impact on replication kinetics or infectious titers of HCV strains JFH1 and DBN3a. The impact of HCV on peroxisomal functions was assessed using sub-genomic replicons. An increase of ROS was measured in peroxisomes of replicon-containing cells, correlated with a significant decrease of catalase activity with the DBN3a strain. In contrast, HCV replication had little to no impact on cytoplasmic and mitochondrial ROS, suggesting that the redox balance of peroxisomes is specifically impaired in cells replicating HCV. Our study provides evidence that peroxisome function and morphology are altered in HCV-infected cells.

## 1. Introduction

Hepatitis C is one of the leading causes of viral chronic hepatitis worldwide. According to WHO estimates, some 58 million people are chronically infected with the hepatitis C virus (HCV), causing about 0.3 million deaths every year (WHO, 2022). About 60–80% of people infected with HCV will develop a chronic infection and are at risk of progressive liver disease, including fibrosis, steatosis, cirrhosis and hepatocellular carcinoma. The infection remains asymptomatic until decades after the initial contact with the virus, when symptoms of liver damage appear. About 10 years ago, efficient antiviral treatments were introduced in the clinics, which cure about 95% of treated patients from their infection. Nevertheless, the risk of HCV-associated disease is reduced but not eradicated among patients with fibrosis who achieve sustained virological response following DAA treatment (van der Meer et al., 2017). Moreover, access to diagnosis remains limited, especially in low-income countries, and a vast majority of HCV-infected persons are unaware of their infected status, therefore HCV keeps spreading. Indeed, it is estimated that 1.5 million new infections occur each year worldwide (WHO, 2022). These data indicate that hepatitis C remains a global health problem, with a severe impact on the quality of life of chronically infected people.

HCV is a small enveloped RNA virus that infects human hepatocytes. Its genome encodes a single open reading frame. It is translated into a precursor polyprotein, which is cleaved into 10 mature structural and non-structural (NS) proteins by host and viral proteases (Lindenbach et al., 2007). All HCV proteins are initially inserted in the membrane of the endoplasmic reticulum (ER). A subset of these proteins (NS3/4A, NS4B, NS5A and NS5B), which are involved in the replication of the genomic RNA, induce membrane rearrangements, including single and double membrane vesicles collectively known as ‘membranous web’, in which the HCV genome is replicated (Ferraris et al., 2010; Romero-Brey et al., 2012). The membranous web likely originates from the ER membrane and includes proteins and lipids normally found in the ER or in other sub-cellular compartments in non-infected cells (Tu et al., 1999; Hamamoto et al., 2005; Stone et al., 2007; Tai et al., 2009; Reiss et al., 2011; Khan et al., 2014; Wang et al., 2014). In addition to these morphological changes, HCV infection alters different cellular pathways, notably metabolic pathways such as glucose metabolism, lipid metabolism and oxidative stress (Hui et al., 2003; Negro, 2010; Virzì et al., 2018). HCV infection also induces neutral lipid accumulation in lipid droplets (Filipe and McLauchlan, 2015; Galli et al., 2021). Recently peroxisome defects were also documented in cell culture and patient samples (Lupberger et al., 2019). As a consequence of these metabolic alterations, HCV infection is often associated with high prevalence of hepatic metabolic disorders such as insulin resistance and/or steatosis (Negro, 2010). HCV-induced alterations in cellular metabolism likely originate in interactions engaged between viral proteins and cellular partners. The HCV genome encodes only a limited number of proteins and relies on host factors for many steps of its life cycle. During HCV replication, proviral host factors are recruited to replication complexes by viral proteins (Reiss et al., 2011; Wang et al., 2014). The recruitment of these proteins may in turn alter their normal cellular functions. Thus, a better understanding of these interactions is crucial to deciphering the alterations in cellular metabolism caused by HCV infection, which are at the origin of pathophysiological disorders.

In order to get more insight into cellular proteins that are recruited in the vicinity of HCV replication complexes, we performed a proximity biotinylation screen based on the use of APEX2 (Rhee et al., 2013). Interestingly, we found that peroxisomes are recruited in the vicinity of HCV replication complexes and that their morphology and function are altered in HCV infected cells. Peroxisomes are small membrane-bound organelles with unique functions in the cell metabolism. Notably, they are involved in lipid metabolism and in the regulation of reactive oxygen species (ROS), two pathways that are critical for HCV infection.

## 2. Materials and methods

### 2.1. Chemicals

Dulbecco’s modified Eagle’s medium (DMEM), Dulbecco’s phosphate-buffered saline (D-PBS), OptiMEM and geneticin were purchased from ThermoFischer Scientific. 4,6-Diamidino-2-phenylindole (DAPI) was from Molecular Probes. Mowiol 3–88 was from Calbiochem. TransIT-LT1 was from Mirus Bio. All other chemicals were from Sigma. Biotin-phenol (BP) was synthesized by mixing equimolar amounts of tyramine (from Sigma-Aldrich) and biotin-OSu (D-Biotin N-hydroxysuccinimide ester, from Iris Biotech) in anhydrous dimethylformamide. The mixture was incubated for 2 h at room temperature. BP synthesis was verified by LC–MS, purified by HPLC and lyophilized.

### 2.2. Antibodies

Mouse monoclonal antibodies (mAb) 9E10 against HCV NS5A (Lindenbach et al., 2005) and 486D39 against NS3 were kindly provided by C. M. Rice (The Rockefeller University) and by J.-F. Delagneau (Bio-Rad), respectively. Rabbit anti-PEX14 and anti-PEX5 antibodies were from ProteinTech. Rabbit anti-ACBD5 and anti-PMP-70 antibodies and mouse mAb TUB2.1 anti-β-tubulin were from Sigma. Anti-catalase rabbit mAb was from Cell Signaling Technologies. Mouse mAb14F3.2 anti-PEX3 was from Millipore. Mouse mAb anti-TOM20 was from BD Biosciences. Mouse mAb anti-myc was produced *in vitro* using a MiniPerm apparatus (Heraeus) following the manufacturer’s protocol. All HRP-conjugated and fluorescent A488-, Cy3-, or Cy5-conjugated secondary antibodies and HRP- or Alexa 488-conjugated streptavidin were from Jackson Immunoresearch.

### 2.3. Plasmids

The plasmid pEGFP-ACBD5 (Costello et al., 2017a) was kindly provided by M. Schrader (University of Exeter). The plasmid pcDNA-myc-CD2AP (Monzo et al., 2005) was kindly provided by M. Cormont (Université Côte d’Azur). Plasmids expressing roGFP2 constructs pMF1707, pMF1706 and pMF1762 (Ivashchenko et al., 2011) were kindly provided by M. Fransen (Katholieke Universiteit Leuven) through Addgene. The plasmid pX330-U6-Chimeric_BB-CBh-hSpCas9 (Cong et al., 2013) was a gift from F. Zhang (Addgene plasmid # 42230). To construct the plasmid pmCherry-SKL, synthetic oligonucleotides 5’-GATCTAAGCTTTG-3′ and 5′- AATTCAAAGCTTA-3′ were phosphorylated, annealed and inserted between BglII and EcoRI sites of the plasmid pmCherry-C1 (Clonetech).

### 2.4. Cell culture

Huh-7 (Nakabayashi et al., 1982) and CD81-deficient Huh-7w7 (Rocha-Perugini et al., 2009) cells were cultured at 37°C with 5% CO_2_ in Dulbecco’s modified Eagle’s medium (DMEM) supplemented with 10% heat-inactivated fetal bovine serum (FBS) and glutamax.

### 2.5. Viruses

The viruses used in this study were the original genotype 2a JFH1 strain (Wakita et al., 2005), or a derivative of this strain bearing titer-enhancing mutations and the reconstitution of the A4 epitope in E1 (Goueslain et al., 2010), and the cell culture-adapted genotype 3a DBN3a strain (Ramirez et al., 2016). To generate genomic HCV RNA, plasmids were linearized by XbaI, treated with mung bean nuclease, and used as templates for *in vitro* transcription with the MEGAscript kit from Ambion. *In vitro*-transcribed RNA was delivered to Huh-7 cells by electroporation as previously described (Kato et al., 2003). Viral stocks were obtained as previously reported (Delgrange et al., 2007) and infectious titers were measured by the TCID50 method. For kinetics analyses, infected cells were trypsinized the day before, counted and 1.5×10^5^ cells were dispensed in a P-24 well in 1 mL of medium. The infectious titer was measured after 24 h of culture. For experiments with PEX3-KO and PEX5-KO Huh-7 cells, a Mann–Whitney test was used to determine statistical differences in infectious titers against the Huh-7 control group.

### 2.6. Replicons

The plasmid pSGR-JFH1 encoding a subgenomic replicon (SGR) of the JFH1 strain was obtained from T. Wakita (Kato et al., 2003). The plasmid pSGR-JFH1-GFP encoding an SGR containing the coding sequence of EGFP in domain III of NS5A has been described (Sahuc et al., 2019). The plasmid pSGR-JFH1-APEX2-∆40 encoding an SGR containing the coding sequence of APEX2 in domain III and a deletion in domain II of NS5A has been described (Riva et al., 2021). The plasmid pSGR-JFH1-Rluc expressing a Renilla luciferase (Rluc) has been previously described (Riva et al., 2021). To construct an Rluc-expressing DBN3a replicon, the 3’-UTR of DBN3a was amplified by PCR using primer pair 5’-GCAGGAATTCTAATACGACTCACTATAGCCTGCC-3′ and 5’-TCATACGCGTTGGGCGGCGGATGGTGTTTCTTTTGG-3′ and inserted into EcoRI and MluI sites of pSGR-JFH1-Rluc, yielding the plasmid pSGR-JFH1-Rluc/3’UTR3a. Then, a DNA fragment containing the EMCV IRES and the coding sequence of the N-terminal part of DBN3a NS3 with no KpnI site was assembled by tripartite fusion PCR using primer pairs 5’-CCAGAAGGTACCCCATTGTATGGG-3′ / 5’-TGTGATCGGGGCCATGGTATCATCGTGTTTTTC-3′, 5’-GATGATACCATGGCCCCGATCACAGCATACACCC-3′ / 5’-ATGAAGGTAgCCGACTTGATAGCT-3′, and 5’-AGCTATCAAGTCGGcTACCTTCAT-3′ / 5’-CTGCTCTAGAGCACTCACAGAGAACAACCGAG-3′ and inserted into KpnI and BsiWI sites of pSGR-JFH1-Rluc/3’UTR3a, yielding the plasmid pSGR-JFH1/3a-Rluc/3’UTR3a. Finally, the BsiWI-XbaI fragment of DBN3a was inserted into the same sites of pSGR-JFH1/3a-Rluc/3’UTR3a, yielding the plasmid pSGR-DBN3a-Rluc. The GND mutation was introduced by fusion PCR using primer pairs 5’-AGGTCGACTCTAGACATGATCTGC-3′ / 5’-TTTCTTGTCTGCGGAaATGATCTG-3′ and 5’-CACCACGACCAGATCATtTCCGCA-3′ / 5’-ATGTGGACCTCAAAGAAGACCCCC-3′. The PCR product was digested with NotI and XbaI and inserted at the corresponding sites in pSGR-DBN3a-Rluc. To construct a DBN3a-based SGR expressing a neomycin resistance marker, the coding sequence of neomycin phosphotransferase was obtained by PCR using the primers 5’-GGGAGAGGGTTTAAACTCAGAAG-3′ and 5’-CCCAACGCGTATGATTGAACAAGATGGATTG-3′, and the PCR product was digested by PmeI and MluI and inserted at the corresponding sites in pSGR-DBN3a-Rluc, yielding the plasmid pSGR-DBN3a. All constructs were verified by sequencing. *In vitro* transcribed SGR RNA was introduced in Huh-7 cells by electroporation (Kato et al., 2006). Electroporated cells were kept in culture for up to 4 days post electroporation for Rluc-expressing SGRs, or were selected using 0.5 mg/mL geneticin to generate Huh-7 cell lines permanently expressing JFH1 or DBN3a SGR.

### 2.7. APEX2-mediated biotinylation

Biotinylation of control and SGR-APEX-containing cells were carried out as previously described (Hung et al., 2016). Briefly, cells were incubated for 30 min at 37°C/5% CO_2_ in DMEM containing 0.5 mM BP. H_2_O_2_ was then added to a final concentration of 1 mM and the cells were incubated for 1 min at room temperature with gentle agitation. The reaction was quenched, and the cells were washed 3 times with D-PBS containing 5 mM Trolox, 10 mM sodium ascorbate and 10 mM NaN_3_ before proceeding to immunoblotting or immunofluorescence.

For purification of biotinylated proteins, Huh-7 cells with or without SGR-APEX were biotinylated as described above. Each cell line was treated with or without H_2_O_2_ after BP incubation. Cell pellets were then lysed in RIPA buffer (50 mM TrisCl pH 7.5, 150 mM NaCl, 0.1% SDS, 0.5% Na deoxycholate, 1% Triton X-100) containing 5 mM Trolox, 10 mM sodium ascorbate and 10 mM NaN_3_ and the lysates were centrifuged at 14,000 x *g*. Streptavidin-coated magnetic beads (Pierce) were washed twice with RIPA buffer, and 2.5 mg of each sample was incubated with 100 μL of magnetic beads slurry with rotation for 2 h at room temperature. The beads were subsequently washed 3 times with RIPA lysis buffer, twice with 1 M KCl, once with 0.1 M Na_2_CO_3_, once with 2 M urea in 10 mM Tris–HCl (pH 8.0), and twice with RIPA lysis buffer. Biotinylated proteins were then eluted from the beads at 95°C for 10 min in 100 μL of 2 X protein loading buffer containing 40 mM DTT and 2 mM biotin.

### 2.8. Generation of knockout cells

Gene invalidation using CRISPR-Cas9 was performed as described (Ran et al., 2013). Briefly, guide RNA coding sequences were ordered as synthetic oligonucleotides, phosphorylated with T4-polynucleotide kinase, annealed and inserted between BbsI sites of plasmid pX330 (Ran et al., 2013). The coding sequences of the guide RNAs were CTCCAGCTGCAAGAAACTCC, AGATGCTGTGGATGTAACTC and CTTGTAGGGGTACCAGTTTG to generate PEX5-KO cells lines and CACCTCCAAGGACCGTGCCC, TAAAGATGCTGAGGTCTGTA and CATATTTCTAGCTTGTTTGA to generate PEX3-KO cells lines. Transfection of Huh-7 cells and selection of KO cell lines were performed as previously described (Ferlin et al., 2018).

### 2.9. Immunofluorescence

Cells were processed for immunofluorescent detection of viral and cellular proteins as previously described (Rouillé et al., 2006). Coverslips were mounted on glass slides using a Mowiol-based medium. For colocalization experiments, confocal microscopy was performed with an LSM880 confocal microscope (Zeiss) using a 63× /1.4 numerical aperture oil immersion objective. Signals were sequentially collected by using single fluorescence excitation and acquisition settings to avoid crossover. For some experiments, high-resolution images (pixel 0.035 × 0.035 μm^2^) were acquired using an Airyscan detector used in SR mode and processed for high-resolution reconstruction using Zen software version 2 (Carl Zeiss Microscopy). Images were assembled by using Adobe Photoshop software. The Pearson’s correlation coefficients (PCC) were calculated using the JACoP plugin of ImageJ software. For each calculation, at least 30 cells were analyzed.

### 2.10. Peroxisome morphology assessment

Huh-7 cells were infected with HCV strains JFH1 or cell culture adapted DBN3a (MOI = 0.1). Infected cells were kept in culture and when they were passaged, approximately 5×10^4^ cells were seeded on glass coverslips. The day after, cells were fixed with 3% PFA, transferred out of the BSL3 laboratory and immunostained as described above using antibodies to NS5A and Pex14. Image stacks of cells of interest were acquired using an inverted Eclipse Ti inverted confocal microscope (Nikon) equipped with a CSU-W1 spinning-disk (Yokogawa, Roper Scientific). A live-SR module (Gataca Systems) was added to the system to improve the obtained resolutions. Observations were done with a 60× oil immersion objective (Nikon Plan Apo 60× NA 1.4). Signals were sequentially collected by using single fluorescence excitation and acquisition settings to avoid crossover. Image stacks were then processed using the Imaris software to model a 3D-image of peroxisomes. Objects smaller than 0.1 μm were filtered out and the volume of each object was measured, enabling the volume of each peroxisome and the average number of peroxisomes per cell to be calculated. For each measurement, 30 cells from 3 independent infections were analyzed. Statistical differences were determined with a Kruskall-Wallis test.

### 2.11. ROS measurements

Plasmids expressing roGFP2 sensors localized in the cytosol, mitochondria or peroxisomes were transfected into control, SGR2a- and SGR3a-containing Huh-7 cells grown in glass-bottom 4 well μ-slide (Ibidi). At 18 h post-transfection, cells were rinsed three times with sterile PBS and placed in phenol red-free DMEM. As a positive control, H_2_O_2_ was added to naïve Huh-7 cells at a final concentration of 0.4 mM. Two hours later, cells were imaged at 37°C and 5% CO_2_ with an LSM880 confocal microscope (Zeiss) using a 63× /1.4 numerical aperture oil immersion objective. Once the acquisition conditions had been set for each roGFP2 sensor, all cells expressing this sensor were acquired using the same parameters. Images were acquired by sequentially exciting with 405 nm and a 488 nm lasers, and then processed using ImageJ software. A region of interest (ROI) was drawn around the whole cell and for both wavelengths the mean intensity of fluorescence was measured. Ratios of fluorescence intensity obtained with each excitation wavelength (405/488) were then calculated. A Kruskall-Wallis test was used to determine statistical differences.

### 2.12. Immunoblotting

Cells were lysed in 50 mM Tris-Cl buffer (pH 7.5) containing 100 mM NaCl, 1 mM EDTA, 1% Triton X-100, 0.1% sodium dodecyl sulfate (SDS), and protease inhibitors for 20 min on ice. Cells were collected, and the nuclei were pelleted. Protein concentration in post-nuclear supernatants was determined by the bicinchoninic acid (BCA) method as recommended by the manufacturer (Sigma), using bovine serum albumin as a standard. Proteins were separated by SDS-polyacrylamide gel electrophoresis and transferred to nitrocellulose membranes (Hybond-ECL; Amersham) by using a Trans-Blot apparatus (Bio-Rad). Blocking was carried out in PBS containing 5% dried skim milk and 0.1% NP-40. Proteins of interest were revealed with specific primary antibodies, followed by species-specific secondary antibodies conjugated to HRP, and enhanced chemiluminescence detection (Super Signal West Pico), as recommended by the manufacturer (ThermoFischer Scientific). The signals were recorded using a LAS 3000 apparatus (Fujifilm). For quantification, unsaturated signals were measured using the gel quantification function of ImageJ (Fiji).

### 2.13. Catalase activity

Catalase activity was measured using a colorimetric catalase assay kit, as recommended by the supplier (Abcam), on cell lysates and normalized by protein content, measured by the BCA method (Sigma).

### 2.14. Lipidomics

For extraction, cells were solubilized in 1 mL of Water/EGTA 5 mM:Methanol (1:2, v/v). Lipids corresponding to total fatty acids in cells were extracted according to Bligh and Dyer (1959) in dichloromethane/water/methanol 2% acetic acid (2.5:2.5:2, v/v/v), in the presence of the internal standards glyceryl trinonadecanoate (4 μg). Extracts were centrifuged during 6 min at 2500 rpm. The lipid extract was hydrolyzed in KOH (0.5 M in methanol, 1 mL) at 55°C for 30 min. A second extraction was realized with 1.5 mL of Methanol, 2 mL of water, and 2.5 mL of dichloromethane. The lipids extract was dried under nitrogen and derivatized with PentafluoroBenzylBromide in ACN (1%) and Diisopropylethylamine in ACN (1%) (1/1, v/v). Final extracts (1 μL) were analyzed on a gas chromatography Thermo Trace 1,310 with mass spectrometer Thermo TSQ 8000 EVO. Chemical ionization is used as ionization, helium as carrier gas, and a HP-5MS column (30 m x 0.25 mm, 0.25 μm) is used to separation. The oven is set at 180°C during 1 min. Then, the temperature is increased at 315°C at 6°C/min during 2 min. SIM method is used to detect the component (m/z 255 to C16:0, m/z 297 to internal standard and m/z 395 to C26:0) and the quantification was obtained through external calibration curves.

### 2.15. Mass spectrometry

APEX2-biotinylated proteins were loaded on SDS-PAGE to perform one gel slice trypsin digestion for each sample. Peptides were extracted with 0.1% formic acid in acetonitrile, evaporated to reduce volume at 8 μL and injected on an UltiMate 3,000 RSLCnano System (Thermo Fisher Scientific). Peptides were automatically fractionated onto a commercial C18 reversed-phase column (75 μm × 250 mm, 2-μm particle, PepMap100 RSLC column, Thermo Fisher Scientific (Waltham, USA), temperature 35°C). Trapping was performed for 4 min at 5 μL/min with solvent A (98% H_2_O, 2% acetonitrile and 0.1% formic acid). Elution was performed using two solvents, A (0.1% formic acid in water) and B (0.1% formic acid in acetonitrile), at a flow rate of 300 nL/min. Gradient separation was 3 min at 3% B, 110 min from 3 to 20% B, 10 min from 20 to 80% B, and maintained for 15 min. The column was equilibrated for 6 min with 3% buffer B prior to the next sample analysis. The eluted peptides from the C18 column were analyzed by Q-Exactive instruments (Thermo Fisher Scientific, Waltham, USA). The electrospray voltage was 1.9 kV, and the capillary temperature was 275°C. Full mass spectrometry (MS) scans were acquired in the Orbitrap mass analyzer over the m/z 400–1,200 range with a resolution of 70,000 (m/z 200). The target value was 3.00E+06. The 15 most intense peaks with charge states between 2 and 5 were fragmented in the high-energy collisional dissociation cell (HCD) with a normalized collision energy of 27%, and the tandem mass spectrum was acquired using an Orbitrap mass analyzer with a resolution of 17,500 at m/z 200. The target value was 1.00E+05. The ion selection threshold was 5.0E+04 counts, and the maximum allowed ion accumulation times were 250 ms for full MS scans and 100 ms for tandem mass spectra. The dynamic exclusion was set to 30 s. The full proteome analysis was performed in same conditions with a column of 500 mm length, heated at 55°C and a 3 to 20% B gradient separation of 170 min. The mass spectrometry proteomics data have been deposited to the ProteomeXchange Consortium via the PRIDE (Perez-Riverol et al., 2022) partner repository with the dataset identifier PXD044994 and 10.6019/PXD044994.

### 2.16. Proteomic data analysis

Raw data collected during nano LC–MS/MS analyses were processed and converted into an *.mgf peak list format with Proteome Discoverer 1.4 (Thermo Fisher Scientific). MS/MS data were analyzed using the search engine Mascot (version 2.4.0, Matrix Science, London, United Kingdom) installed on a local server. Searches were performed with a tolerance on mass measurement of 0.02 Da for precursor and 10 ppm for fragment ions against a composite target-decoy database built with a Human Swissprot database (taxonomy 9,606) fused with the sequences of recombinant trypsin, NS3, NS4A, NS4B, NS5A, NS5B, and a list of classical contaminants. Cysteine carbamidomethylation, methionine oxidation, protein N-terminal acetylation, and cysteine propionamidation were searched as variable modifications. Up to one missed trypsin cleavage was allowed.

For the APEX2-biotinylated protein samples, the identification results were imported into Scaffold software 4.5.3 and peptides were filtered out according to the cutoff set for proteins hits with 1 or more peptides taller than 9 residues, ion score > 20, identity score > −7, corresponding to a 1% false positive rate.

For the full proteome analysis, the identification results were imported into Proline software^1^ (Bouyssié et al., 2020). Peptide spectrum matches taller than 9 residues and ion scores >10 were retained. The false discovery rate was then optimized to be below 1% at the protein level using the Mascot Modified Mudpit score. Extracted Ion Current based quantification was performed with Proline 2.0. At peptide level, a single charge level per peptide was selected. Then, for peptides with 0 spectra, those with abundance above the upper bound of the 99% confidence interval of the distribution were filtered out. The abundance of a protein was obtained by summing the abundances of its component peptides.

Statistical analysis was performed by using R (version 4.1.2). Data were log2 transformed and distributions were centered on the upper quartile. Only proteins with at least three measurements (non missing values) in at least one group were kept. Following recommendations of Tyanova et al. (2016), missing values were then imputed using a normal distribution *X* ~ (*μ*-1.8*σ*, 0.3*σ*). Differential expression analysis between control Huh-7 and SGR-JFH1 and between control Huh-7 and SGR-DBN3a was performed using limma R package (Ritchie et al., 2015), which uses an empirical Bayesian approach to estimate variances in moderated t tests. Raw *p* values were adjusted for multiple testing using the Benjamini–Hochberg procedure (Benjamini and Hochberg, 1995). Proteins were considered differentially expressed if their adjusted *p*-values were below 0.05.

In order to evaluate the ability of peroxisomal proteins to discriminate groups when considered altogether, a multivariate analysis was performed with the mixOmics R package, using the partial least squares – discriminant approach (PLS-DA) (Rohart et al., 2017). Only the peroxisomal proteins were kept in this multivariate analysis.

## 3. Results

### 3.1. Peroxisomes are recruited close to HCV replication complexes

**Figure 1:**
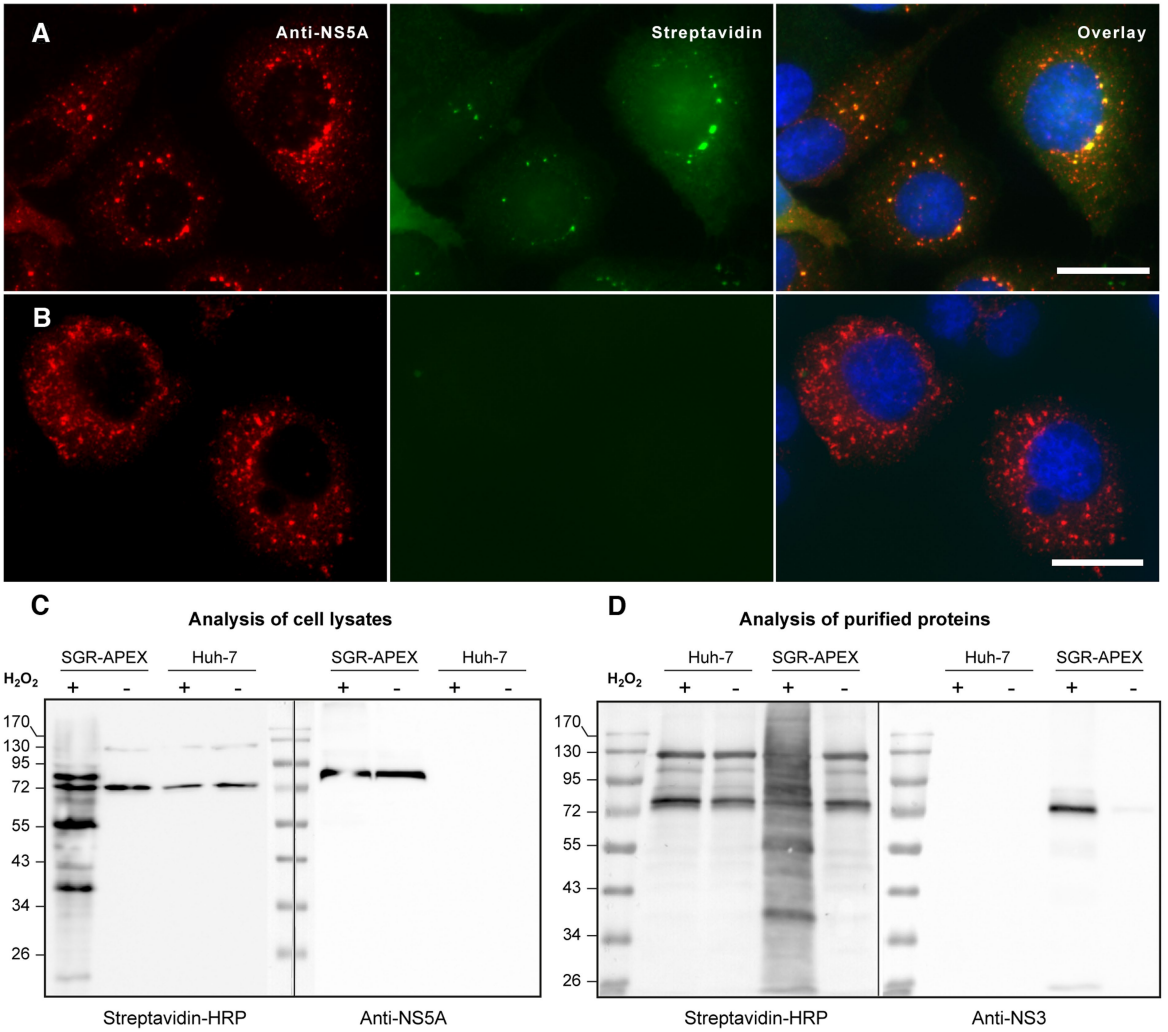
**(A,B)** Immunofluorescence microscopy of biotinylated cells expressing the SGR-APEX **(A)** or non-tagged SGR **(B)** double labeled with A488-streptavidin (green) and with anti-NS5A antibody (red). Nuclei were stained with DAPI (blue). Bars, 20 μm. **(C)** Immunoblot analysis of biotinylated proteins. HCV SGR-APEX-containing and control Huh-7 cells pre-incubated in the presence of BP were treated in the presence (+) or in the absence (−) of H_2_O_2_ for 1 min, quenched and lysed. Cell lysates were probed with streptavidin-HRP (left side of the blot) or with an anti-NS5A antibody (right side of the blot). **(D)** Immunoblot analysis of proteins purified with strepatividin beads. HCV SGR-APEX-containing and control Huh-7 cells were processed as described in **(C)**. Biotinylated proteins were purified using strepatvidin beads and analyzed by immunoblot with streptavidin-HRP (left side of the blot) and with an antibody to HCV NS3 (right side of the blot). Note that mammalian cells have four endogenously biotinylated proteins that are detected in control conditions.

To identify cellular proteins associated to HCV replication complexes, we used an SGR of JFH1 strain containing the small peroxidase APEX2 inserted into domain III of NS5A (Riva et al., 2021). The catalytic activity of APEX2 was used to biotinylate proteins that are in close proximity (less than 20 nm) to the NS5A-APEX2 fusion protein using BP as a substrate (Rhee et al., 2013). Cells were incubated with APEX2 substrates BP and H_2_O_2_ for 1 min and the presence of biotinylated material was verified by immunofluorescence and immunoblotting. SGR-APEX-containing cells displayed double-labeled dot-like structures when they were probed with Alexa488-conjugated streptavidin to reveal the localization of biotinylated material and with an anti-NS5A antibody as a marker of HCV replication complexes (Figure 1A). These double-labeled structures were found in SGR-APEX-containing, but not in non-tagged SGR-containing Huh-7 cells (Figure 1B). When the biotinylated proteins were analyzed by immunoblot, several specific bands were detected in SGR-containing cells incubated with APEX2 substrates (Figure 1C, left blot), in addition to a few bands present in control samples, which correspond to cellular proteins that are endogenously biotinylated. When the same samples were analyzed with an anti-NS5A antibody, the NS5A-APEX2 fusion protein was detected at a molecular size very similar to that of a major band of the biotinylated protein (Figure 1C, right blot), indicating that, as expected, NS5A-APEX2 was one of the most efficiently biotinylated proteins.

Biotinylated proteins were purified with streptavidin-coupled agarose beads. Streptavidin blot analysis of the eluted proteins revealed a large range of biotinylated proteins, whereas the controls again showed only endogenously biotinylated proteins (Figure 1D, left blot). The same membrane was then re-probed with an anti-NS3 antibody to verify that a known component of replication complexes was actually biotinylated. As expected, HCV NS3 was detected among the biotinylated proteins purified from the lysate of SGR-containing cells incubated in the presence of APEX2 substrates, but not under control conditions with no substrate and/or no peroxidase (Figure 1D, right blot). These results validate the use of this method to biotinylate and purify constituent proteins of HCV replication complexes.

Next, we analyzed the proteins purified after APEX2-mediated biotinylation in SGR-containing cells. Affinity purified proteins derived from biotinylated and non-biotinylated control and SGR-APEX-containing Huh-7 cells were subjected to mass spectrometry analysis. Liquid chromatography tandem-mass spectrometry (LC–MS/MS) was performed from in-gel digested samples and spectral counting was used to compare protein enrichment between biotinylated and non-biotinylated SGR-APEX samples. Forty-eight cellular proteins were identified with at least 2 unique peptides in the biotinylated SGR-APEX sample, no peptide in the non-biotinylated SGR-APEX sample and in both control Huh-7 samples (Table 1). Of these 48 proteins, CD2AP (Igloi et al., 2015; Zhang et al., 2018) and PACSIN2 (Nguyen et al., 2020) have previously been shown to interact with NS5A and Scribble with NS4B (Hu et al., 2016) in HCV-infected cells. Another host factor potentially recruited to HCV replication complexes is Ago2, which interacts with HCV RNA 5’-UTR in association with miR122 (Shimakami et al., 2012). In addition, 11 other proteins have been reported to interact with NS5A and 5 proteins with NS3, NS4B or NS5B in proteomics or yeast two-hybrid screens (de Chassey et al., 2008; Dolan et al., 2013; Germain et al., 2014; Ramage et al., 2015; Goonawardane et al., 2017), but their recruitment to HCV replication complexes has not been established. It should be noted that proteins identified with this approach include proteins that are very close to each other, without necessarily being interactors.

**Table 1:** Proteins identified in the proximity biotinylation screen.

Protein name	Accession number	Size (Da)	Unique peptides				coverage
Sample Biotinylation			SGR		Hu		
			+	-	+	-	
CD2-associated protein	Q9Y5K6	71,454	16	0	0	0	32%
Acyl-CoA-binding domain-containing protein 5	Q5T8D3	60,092	6	0	0	0	12%
Sorbin and SH3 domain-containing protein 1	Q9BX66	142,517	5	0	0	0	6%
Filamin-B	O75369	278,159	6	0	0	0	3%
Protein kinase C and casein kinase substrate in neurons protein 2	Q9UNF0	55,739	5	0	0	0	12%
Serine/threonine-protein kinase Nek9	Q8TD19	107,170	4	0	0	0	6%
Arsenite methyltransferase	Q9HBK9	41,748	3	0	0	0	10%
Hepatocyte growth factor-regulated tyrosine kinase substrate	O14964	86,192	2	0	0	0	3%
Protein transport protein Sec16A	O15027	233,515	3	0	0	0	2%
Vinexin	O60504	75,342	3	0	0	0	8%
Peroxisomal membrane protein PEX14	O75381	41,237	2	0	0	0	6%
Integrin beta-1	P05556	88,415	3	0	0	0	5%
Alpha actinin-1	P12814	103,061	2	0	0	0	3%
Nuclear factor NF-kappa-B p100 subunit	Q00653	96,751	3	0	0	0	4%
Deubiquitinating protein VCIP135	Q96JH7	134,322	3	0	0	0	4%
Tumor susceptibility gene 101 protein	Q99816	43,946	3	0	0	0	9%
NSFL1 cofactor p47	Q9UNZ2	40,573	3	0	0	0	14%
Protein phophatase 1 regulatory subunit 12A	O14974	115,283	2	0	0	0	2%
U2 snRNP-associated SURP motif-containing protein	O15042	118,296	2	0	0	0	3%
Heterogeneous nuclear ribonucleoprotein R	O43390	70,944	2	0	0	0	4%
Transforming acidic coiled-coil-containing protein 1	O75410	87,796	2	0	0	0	4%
NADH dehydrogenase [ubiquinone] 1 beta subcomplex subunit 4	O95168	15,210	2	0	0	0	22%
Integrin alpha-2	P17301	129,298	2	0	0	0	2%
Heterogeneous nuclear ribonucleoproteins A2	P22626	37,430	2	0	0	0	8%
Nuclear pore complex protein Nup214	P35658	213,619	2	0	0	0	1%
Peripherin	P41219	53,652	2	0	0	0	4%
E3 ubiquitin-protein ligase NEDD4	P50402	149,118	2	0	0	0	2%
Emerin	P50402	28,995	2	0	0	0	9%
14–3-3 protein gamma	P61981	28,303	2	0	0	0	10%
Focal adhesion kinase 1	Q05397	119,236	2	0	0	0	3%
Golgin subfamily A member 2	Q08379	113,086	2	0	0	0	2%
Protein scribble homolog	Q14160	174,887	2	0	0	0	1%
Proteasome activator complex subunit 4	Q14997	211,341	2	0	0	0	2%
Leucine-rich repeat flightless-interacting protein 1	Q32MZ4	89,253	2	0	0	0	4%
Striatin-interacting protein 1	Q5VSL9	95,579	2	0	0	0	2%
Ankyrin repeat and LEM domain-containing protein 2	Q86XL3	104,117	2	0	0	0	4%
Cell division cycle and apoptosis regulator protein 1	Q8IX12	132,824	2	0	0	0	2%
Proline-, glutamic acid- and leucine-rich protein 1	Q8IZL8	119,701	2	0	0	0	2%
EH domain-binding protein 1	Q8NDI1	140,021	2	0	0	0	2%
Pre-B-cell leukemia transcription factor-interacting protein 1	Q96AQ6	80,643	2	0	0	0	3%
Dedicator of cytokinesis protein 7	Q96N67	242,566	2	0	0	0	1%
182 kDa tankyrase-1-binding protein	Q9C0C2	181,795	2	0	0	0	2%
Tyrosine-protein phohatase non-receptor type 23	Q9H3S7	178,975	2	0	0	0	2%
Exocyst complex component 1	Q9NV70	101,984	2	0	0	0	2%
Prefoldin subunit 2	Q9UHV9	16,648	2	0	0	0	17%
Protein argonaute-2	Q9UKV8	97,208	2	0	0	0	3%
Melanoma-associated antigen D2	Q9UNF1	64,956	2	0	0	0	3%
Nuclear migration protein nudC	Q9Y266	38,244	2	0	0	0	8%

We verified by immunofluorescence microscopy the recruitment to HCV-replication complexes of four out of the top five proteins in Table 1, which correspond to proteins identified with a spectral ratio at least equal to 10. Tagged constructs of these proteins were expressed in control and SGR-containing Huh-7 cells, and their intracellular localization was compared to that of HCV NS5A in SGR-containing cells. CD2-associated protein (CD2AP), a protein of the plasma membrane, was massively recruited to NS5A-positive dot-like structures (Supplementary Figure S1), as expected (Igloi et al., 2015; Zhang et al., 2018). The intracellular distribution of Acyl-CoA-binding domain-containing protein 5 (ACBD5, a peroxisomal membrane protein) was also altered in SGR-containing cells, as compared to control Huh-7 cells (Supplementary Figure S2). In contrast, no visible change of intracellular localization was observed for Protein kinase C and casein kinase substrate in neurons protein 2 (PACSIN2, also known as syndapin II), or for filamin B (data not shown). The reason for this apparent absence of recruitment is unclear, but it could represent proteins for which only a minor fraction of the cellular pool may associate with HCV replication complexes. Alternatively, they may represent proteins interacting with a pool of NS5A located in the ER membrane, outside the replication complexes.

**Figure 2:**
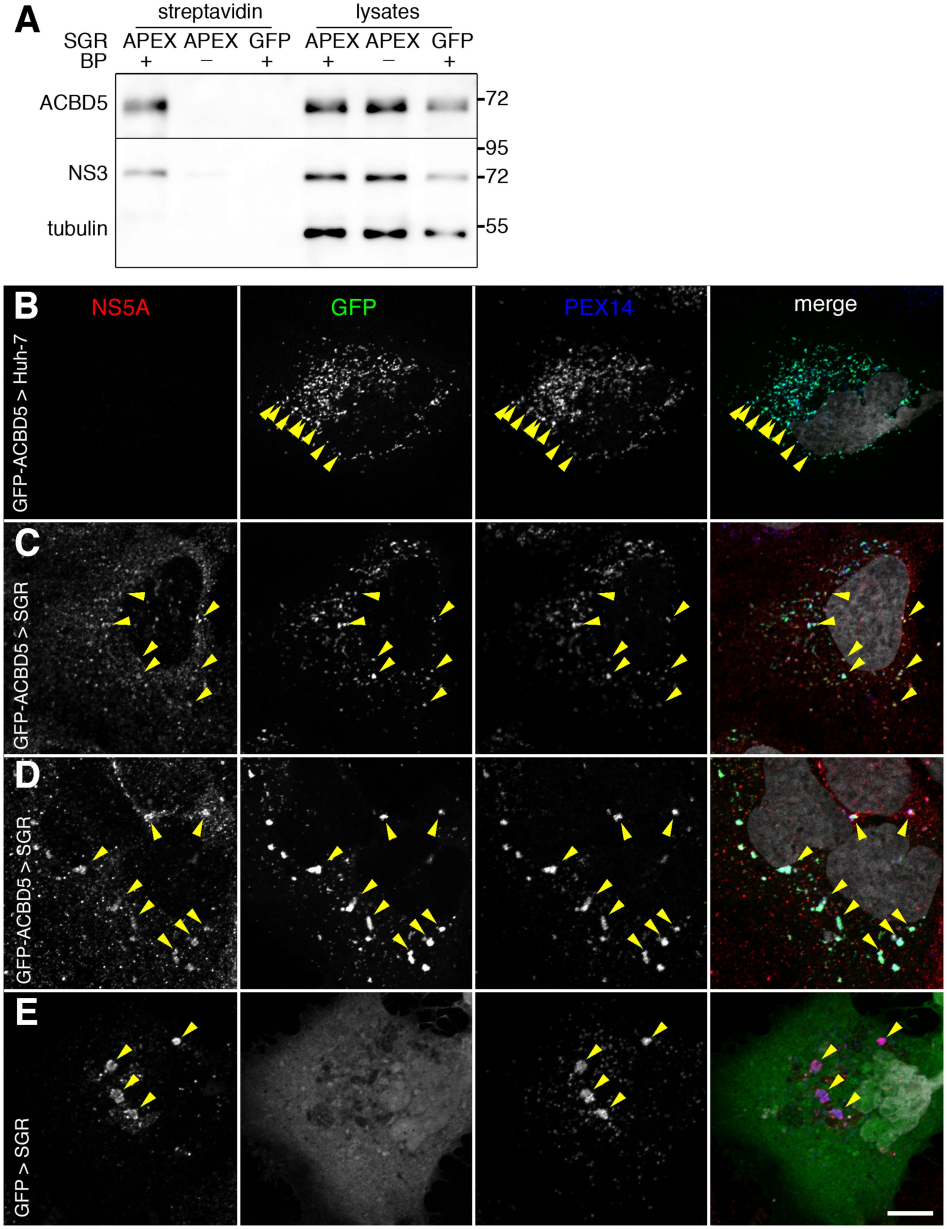
**(A)** APEX2-mediated biotinylation of ACBD5. Huh-7 cells containing APEX2- or GFP-tagged HCV replicons (SGR) were pre-incubated in the presence (+) or the absence (−) of BP, treated with H_2_O_2_ for 1 min, quenched and lysed in RIPA buffer. Biotinylated proteins were purified using strepatvidin beads and analyzed by immunoblot, together with 8% of the inputs (lysates), with antibodies to ACBD5, HCV NS3 and tubulin. **(B–E)** Intracellular localization of GFP-ACBD5 transfected in naïve Huh-7 cells **(B)** or in HCV SGR-containing Huh-7 cells **(C,D)**. As a control, GFP was expressed in SGR-containing cells **(E)**. Transfected cells were fixed and processed for immunofluorescence staining of HCV NS5A (left panels, red in merge panels) and PEX14 (third panels from the left, blue in merge panels), and imaged with a confocal microscope. GFP-ACBD5 and GFP (second panels from the left, green in merge panels) were detected using GFP fluorescence and nuclei were stained with DAPI (grey in merge panels). Yellow arrowheads indicate examples of regions where markers are co-distributed. Bar, 10 μm.

Interestingly, in addition to ACBD5, another peroxisome membrane protein, PEX14, was present in the list of proteins identified in this screen (Table 1). This suggested that peroxisomes could be present in the vicinity of HCV replication complexes. To confirm that ACBD5 was actually biotinylated in cells with an APEX2-containing SGR, biotinylated proteins were analyzed by immunoblotting using an anti-ACBD5 antibody. ACBD5 was identified in the pool of proteins biotinylated by APEX2 in the presence of both of its substrates, but not in the absence of BP or if APEX2 was replaced by GFP in the replicon (Figure 2A). This result confirmed that ACBD5 is actually biotinylated in cells containing an APEX2-SGR, and suggested that it could be recruited in the proximity of HCV-replication complexes.

To test whether ACBD5 relocalized from peroxisomes to HCV replication complexes or whole peroxisomes were recruited to the proximity of HCV-replication complexes, we expressed a GFP-tagged version of ACBD5 in naïve or SGR-containing Huh-7 cells and labeled them with antibodies to NS5A, a marker of HCV replication complexes, and PEX14, a marker of peroxisomes. In both naïve and SGR-containing cells, GFP-ACBD5 was co-distributed with PEX14, indicating that it was correctly localized in peroxisomes in naïve (Figure 2B) and SGR-containing cells (Figures 2C,D). The localization of GFP-ACBD5 to peroxisomes was supported by Pearson correlation coefficients of 0.80 ± 0.07 measured in SGR-containing cells and 0.81 ± 0.07 in control Huh-7 cells. In SGR-containing cells, GFP-ACBD5 was also associated with NS5A-positive structures (Figures 2C,D), confirming the recruitment of peroxisomes to the proximity of HCV-replication complexes. It is worth noting that the proximity of peroxisomes and HCV-replication complexes was not induced by GFP-ACBD5 expression, because a colocalization of NS5A and PEX14 was also observed in GFP-transfected cells (Figure 2E).

### 3.2. Alteration of peroxisomes morphology in replicon-containing Huh-7 cells

**Figure 3:**
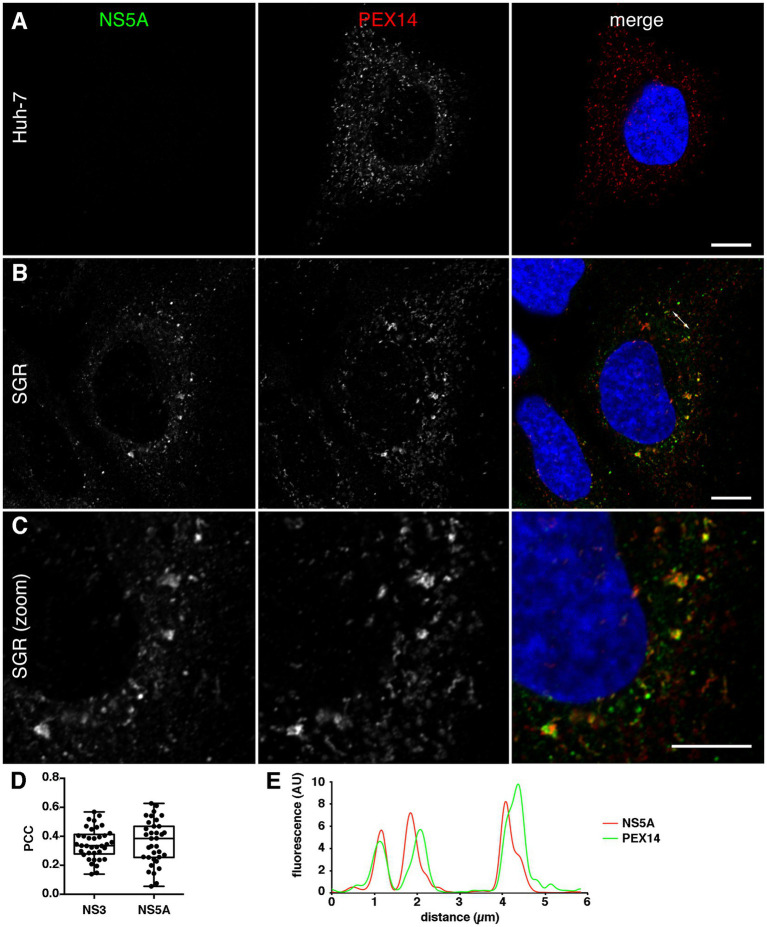
**(A–C)** Intracellular localization of endogenous PEX14 in naïve **(A)** or HCV SGR-containing Huh-7 cells **(B,C)**. Cells were fixed and processed for immunofluorescence staining of HCV NS5A (left panels, green in merge panels) and PEX14 (middle panels, red in merge panels), and imaged with a high-resolution (Airyscan) confocal microscope. Nuclei were stained with DAPI (blue in merge panels). Bars, 10 μm. **(D)** Analysis of ACBD5 colocalization with HCV NS3 and NS5A. HCV SGR-containing Huh-7 cells were double labeled with antibodies to ACBD5 and NS3 or NS5A, and the Pearson correlation coefficient (PCC) was calculated on at least 30 individual cells imaged with a confocal microscope. **(E)** Fluorescence intensity plot along the double arrowed line indicated in **B**, merge panel.

Interestingly, not only the localization of peroxisomes but also their morphology appeared to be altered in a fraction of JFH1 SGR-containing cells expressing GFP-ACBD5 (Figure 2D). To assess if the change of peroxisome morphology was induced or not by GFP-ACBD5 expression in transfected cells, we analyzed it in untransfected SGR-containing cells using PEX14 as an endogenous marker. Again, a partial relocalization of peroxisomes in the vicinity of HCV replication complexes was observed (Figure 3B). When compared with peroxisomes of control cells (Figure 3A), larger peroxisomes were present in some but not all SGR-containing cells. Altered peroxisomal structures included both larger structures and long thin tubular structures. Cells displaying peroxisomes with an altered morphology were also observed in cells containing a sub-genomic replicon of strain DBN3a (data not shown). These altered peroxisome structures were not always associated with NS5A, suggesting that the alteration of their morphology does not directly depend on their association with HCV replication complexes. In other cells, near-normal sized peroxisomes colocalize with HCV replication complexes, again indicating that the morphological alteration of peroxisomes does not uniquely depend on their association with HCV replication complexes.

### 3.3. Association of peroxisomes and HCV-replication complexes

The association of peroxisomes and HCV replication complexes could also be observed using ACBD5 as an endogenous peroxisome marker (Supplementary Figure S3A) and in cells expressing mCherry-SKL, a red fluorescent peroxisomal marker (Supplementary Figure S3B). In any case, peroxisomes and HCV replication complexes often appeared juxtaposed rather than completely colocalized, when visualized at high resolution (Figure 3C). Pearson correlation coefficient values obtained when the co-distribution of ACBD5 and NS5A or NS3 was analyzed (Figure 3D) are consistent with a partial colocalization or a close proximity of peroxisome and HCV replication complex markers. Fluorescence intensity plots also indicated that the two markers are close from one another but not colocalized (Figure 3E).

ACBD5 interacts with VAP-A and VAP-B via a FFAT motif and has been reported to be involved in the interaction between peroxisomes and the membrane of the endoplasmic reticulum (Hua et al., 2017; Costello et al., 2017a). Since VAP-A and VAP-B also interact with NS5A in HCV-infected cells and are recruited to HCV-replication complexes (Tu et al., 1999; Hamamoto et al., 2005), we investigated whether ACBD5 could be involved in the association of peroxisomes and HCV replication complexes through its interaction with VAP-A/B. The gene encoding ACBD5 was inactivated by CRISPR-Cas9 technology (Supplementary Figure S4) and a HCV SGR was introduced in ACBD5-KO cells. No difference of replication efficiency was observed between ACBD5-KO and control Huh-7 cells (data not shown), indicating that ACBD5 is not essential for HCV replication. No major differences in peroxisome phenotypes regarding both association to HCV-replication complexes and morphological alterations was observed in ACBD5-KO cells as compared to control Huh-7 cells (Supplementary Figure S4). This result suggests that ACBD5 is not involved in peroxisome association to HCV replication complexes. Alternatively, ACBD5 could be part of a complex involved in this association, but its inactivation would not be sufficient to inhibit it.

### 3.4. Alteration of peroxisomes morphology in HCV-infected cells

**Figure 4:**
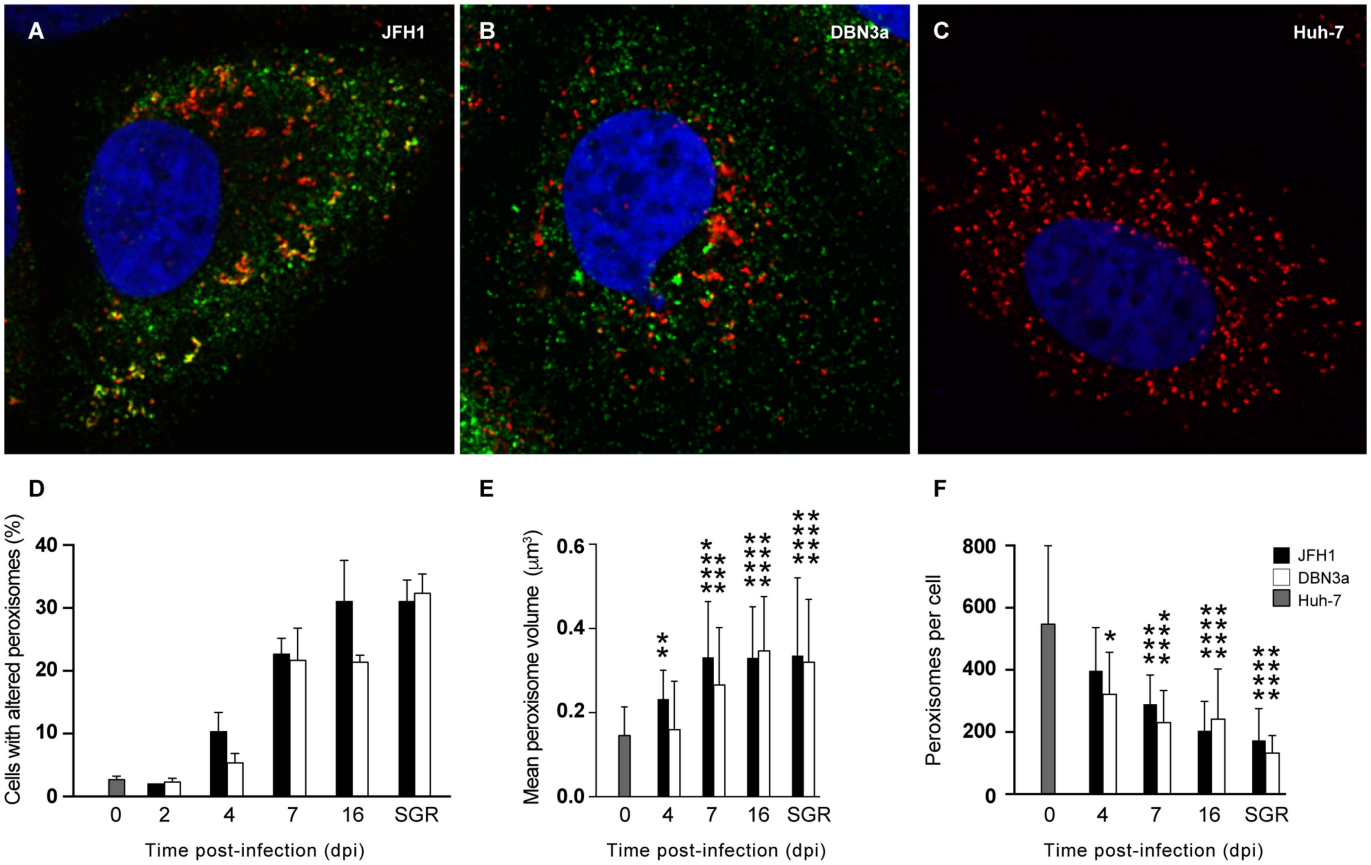
Alteration of peroxisome morphology in HCV-infected and replicon-containing Huh-7 cells. **(A–C)** Immunofluorescence analysis of NS5A (green) and PEX14 (red) at 7 days after infection with JFH1 (A) or DBN3a **(B)** strains, or in naïve Huh-7 cells **(C)**. **(D)** Number of cells with altered (larger and/or tubule-like) peroxisomes in HCV infected cells at different time points post infection and in Huh-7 cells permanently expressing a JFH1 or DBN3a SGR. **(E)** Mean volume of peroxisomes. **(F)** Mean number of peroxisomes per cell. Bars represent the mean +/− SD of 30 cells from 3 different infections. *, *p* < 0.05; **, *p* < 0.01; ***, *p* < 0.001; ****, *p* < 0.0001 (Kruskall-Wallis test).

Next, we investigated peroxisome morphology in HCV-infected cells. Huh-7 cells were infected with HCV strains JFH1 and DBN3a and fixed at different dpi to analyze peroxisome morphology by immunofluorescence microscopy. For this series of experiments, we used the culture adapted DBN3a strain and the original, non-adapted JFH1 strain, because the cell culture-adapted JFH1 strain did not allow to keep infected cells for up to 16 dpi. Like in SGR-containing cells, some infected Huh-7 cells displayed peroxisome/replication complex association and altered peroxisome morphology for both strains (Figures 4A–C). The number of cells displaying altered peroxisomes increased over time and reached 20–30% at 16 dpi, a number similar to what was observed in cells permanently expressing an SGR (Figure 4D).

We characterized these morphological alterations using confocal microscopy and 3D-reconstitution in naïve and infected cells over time. The average volume of a peroxisome increased steadily from 0.15 ± 0.01 μm^3^ in naïve Huh-7 cells to 0.35 ± 0.02 μm^3^ at 16 dpi in DBN3a-infected cells and to 0.33 ± 0.02 μm^3^ in JFH1-infected cells (Figure 4E). At the same time, the average number of peroxisomes per cell dropped from 547 ± 94 in naïve Huh-7 cells to 242 ± 59 in DBN3a-infected cells and 204 ± 95 in JFH1-infected cells (Figure 4F). As a consequence of this increase in mean volume coupled to a decrease in peroxisome number per cell, the total volume occupied by peroxisomes in a cell displaying altered peroxisome morphology did not significantly change over time from their total volume in non-infected cells.

### 3.5. Analysis of peroxisomal proteins expression by mass spectrometry

**Figure 5:**
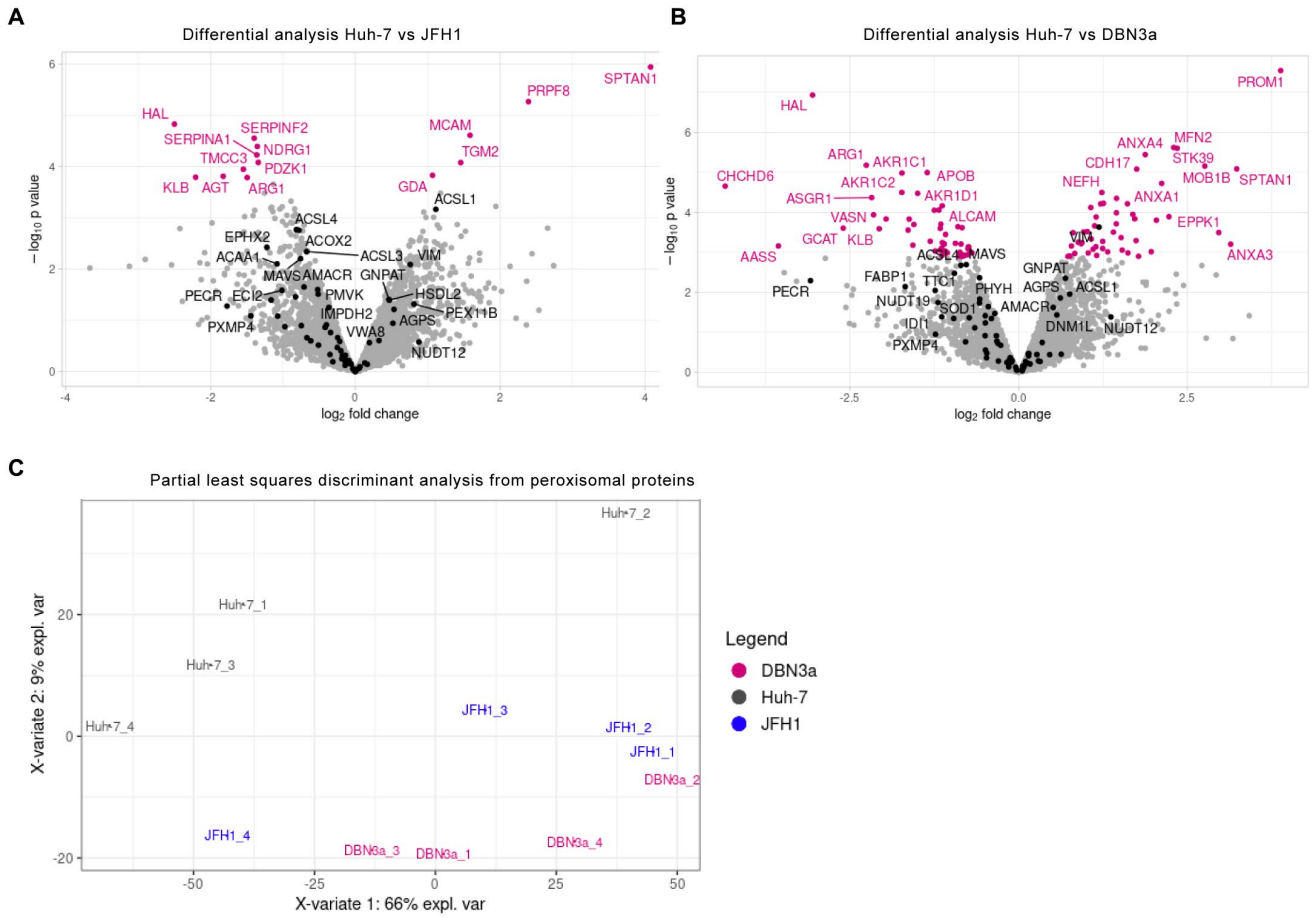
Differential expression analysis of proteins in JFH1 **(A)** and DBN3a **(B)** SGR-containing cells. Proteins of total cell lysates were analyzed by LC–MS/MS (4 replicates per sample). Peroxisome proteins are displayed in black and other identified proteins either in pink (those with a significant difference of expression) or in grey (non differentially expressed). **(C)** Multivariate analysis of peroxisomal proteins expression using PLS-DA (Partial Least-Squares Discriminant Analysis). Groups to be discriminated were control Huh-7 cells (Hu) and DBN3a and JFH1 SGR-containing cells.

To further assess if these morphological alterations are linked to biochemical alterations of peroxisomes, their protein content was analyzed by mass spectrometry. Total cellular proteins of control Huh-7 cells, of JFH1 replicon-containing cells and of DBN3a replicon-containing cells (50 μg per sample, 4 samples per cell line) were subjected to analysis. Overall, 4,072 proteins were identified, and the expression levels of proteins identified in at least 3 samples from at least one cell line (3,768 proteins), of which 63 proteins with a GO annotation: peroxisome, were used to compare protein expression between samples. Overall, 14 proteins were found to be differentially expressed in JFH1 replicon-containing cells (Figure 5A) and 97 proteins in DBN3a replicon-containing cells (Figure 5B), of which the only protein with a “peroxisome” GO annotation was vimentin in the DBN3a replicon. None of the peroxisomal membrane and matrix proteins was dysregulated in a significant manner. These data indicate that the variation of expression of peroxisomal proteins in SGR-containing cells is too moderate to be appreciated by differential analysis with only four replicates per group. In particular, some proteins, such as PECR for example, show a fold change comparable to differentially expressed proteins (Figure 5B) but the variability between replicates is too high to observe an adjusted value of p below 0.05. Moreover, these differential analyses were performed protein by protein. We observed that multivariate analyses separated Huh-7 from DBN3a and JFH1 when looking at only peroxisome proteins (Figure 5C). This suggests that even if individual peroxisome proteins present a moderate change of expression levels, HCV replication has a significant effect on their expression when being taken together.

### 3.6. Role of peroxisomes during the HCV life cycle

**Figure 6:**
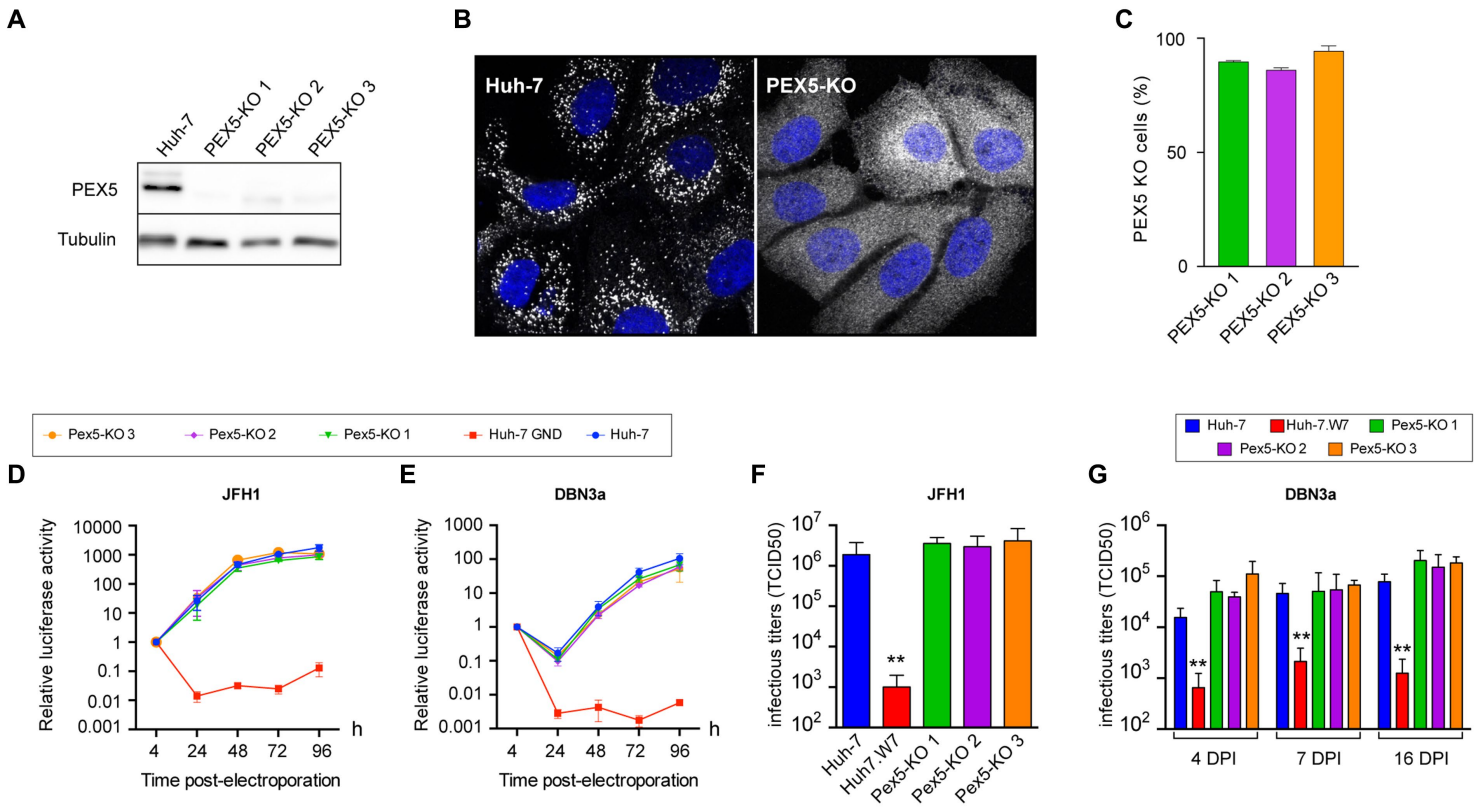
HCV infection in PEX5-KO Huh-7 cells. **(A)** Immunoblot analysis of Pex5 protein expression in control and PEX5-KO cells. **(B)** Immunofluorescence pattern of catalase in control and PEX5-KO cells. **(C)** Quantification of the number of PEX5-KO cells in the 3 populations using the catalase pattern assay. **(D,E)** Huh-7 cells were electroporated with JFH1 SGR **(D)** or a DBN3a SGR **(E)** expressing Renilla luciferase or with a non-replicative SGR (GND). Samples were harvested for luciferase assay at 4, 24, 48, 72, and 96 h post electroporation. The luciferase activity at 4 h post electroporation is expressed as 1. Error bars indicate standard errors of the means for 3 independent experiments performed in quadruplicate samples. **(F)** Huh-7, CD81-deficient Huh-7w7 and PEX5-KO cells were infected with JFH1 and cultured for 3 days. Supernatants were collected, filtered and infectious titers were determined. **(G)** Huh-7, CD81-deficient Huh-7w7 and PEX5-KO cells were infected with DBN3a and passed at 3, 6, and 15 dpi. At each passage, 1.5×10^5^ cells were seeded in a well of a P-24 plate. Twenty-four hours later, supernatants were collected and infectious titers were determined. *, *p* < 0.05; **, *p* < 0.01 (Mann–Whitney test).

To assess the importance of peroxisomes in the HCV life cycle, we generated a series of Huh-7-derived cells with non-functional peroxisomes or no peroxisomes at all. Cells with non-functional peroxisomes were generated by inactivating the PEX5 gene. The Pex5 protein is involved in the import of peroxisomal matrix proteins containing a type 1 peroxisomal targeting signal (PTS1), consisting of a carboxy-terminal -serine-lysine-leucine (-SKL) tripeptide (Ma et al., 2011) and the inactivation of the PEX5 gene leads to non-functional peroxisomes (Baes et al., 1997). Three Huh-7 derived PEX5-KO cell lines were generated with 3 different guide RNAs using the CRISPR-Cas9 technology. These cell lines had less than 5% Pex5 protein expression levels (Figure 6A), as estimated by immunoblotting. Because cells were not cloned after the transfection of the CRISPR-Cas9 plasmid, we can expect that a percentage of them express a functional Pex5 protein, due to the random mode of repair exerted by the cell’s NHEJ pathway after the genomic DNA has been cut by the Cas9 nuclease. Catalase is imported in peroxisomes in a Pex5-dependent manner (Freitas et al., 2011). To evaluate the number of cells with non-functional peroxisomes, we labeled cells by immunofluorescence with an anti-catalase antibody to visualize its intracellular localization. In control cells, catalase pattern of labeling was made of dot-like structures, corresponding to peroxisomes, while this pattern was lost in PEX5-KO cells and catalase appeared merely cytosolic (Figure 6B). Using this assay, the number of cells with no functional peroxisomes was quantified as approximately 90% for the 3 PEX5-KO cell lines (Figure 6C).

Since peroxisomes are partially recruited near replication complexes, we first studied the impact of the absence of functional peroxisomes on HCV replication using luciferase-expressing SGRs of HCV strains JFH1 and DBN3a. We monitored their replication during 4 days and did not observe any significant difference in HCV replication (Figures 6D,E). We also titered infectious particles secreted by PEX5-KO cell lines. Cells were infected and infectious particles were measured in cell culture media at 3 dpi for JFH1. For DBN3a, which is less toxic than the cell culture-adapted JFH1 strain, infectious titers could be measured up to 16 dpi (Figure 4). Huh-7w7 cells, which do not express CD81 (Rocha-Perugini et al., 2009), were used as controls. Again, the absence of functional peroxisomes had no impact on HCV infectious titers (Figures 6F,G). These results indicate that functional peroxisomes are not required for HCV to establish an infection.

**Figure 7:**
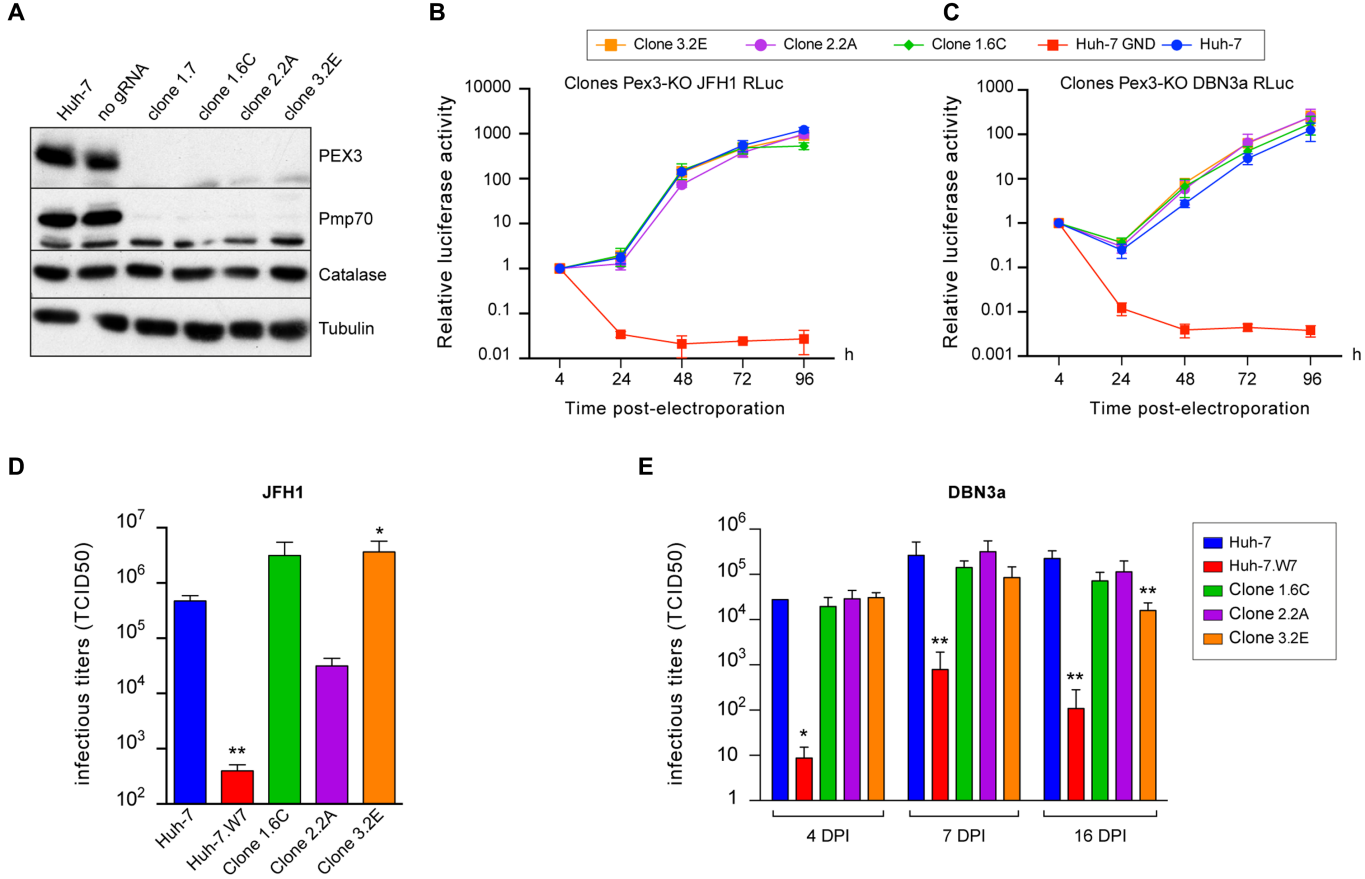
HCV infection in PEX3-KO Huh-7 cells. **(A)** Immunoblot analysis of Pex3 protein, Pmp-70, catalase and tubulin expression in control and selected clones of PEX3-KO cells. **(B,C)** Huh-7 cells were electroporated with JFH1 SGR **(B)** or a DBN3a SGR **(C)** expressing Renilla luciferase or with a non-replicative SGR (GND). Samples were harvested for luciferase assay at 4, 24, 48, 72, and 96 h post electroporation. The luciferase activity at 4 h post electroporation is expressed as 1. Error bars indicate standard errors of the means for 3 independent experiments performed in quadruplicate samples. **(D)** Huh-7, CD81-deficient Huh-7w7 and PEX3-KO cells were infected with JFH1 and cultured for 3 days. Supernatants were collected, filtered and infectious titers were determined. **(E)** Huh-7, CD81-deficient Huh-7w7 and PEX3-KO cells were infected with DBN3a and passed at 3, 6 and 15 dpi. At each passage, 1.5×10^5^ cells were seeded in a well of a P-24 plate. Twenty-four hours later, supernatants were collected and infectious titers were determined. *, *p* < 0.05; **, *p* < 0.01 (Mann–Whitney test).

To further assess the role of peroxisomes during the HCV life cycle, we generated PEX3-KO cell lines. The Pex3 protein is a component of the peroxisome membrane protein insertion machinery (Ma et al., 2011), and its inactivation results in cells completely devoid of peroxisomes (Hettema et al., 2000). Huh-7-derived PEX3-KO cell lines were generated with 3 different guide RNAs. However, only about 50% of cells displayed a catalase pattern consistent with a complete absence of peroxisome. Therefore, we isolated by limited dilution cellular clones completely devoid of peroxisomes using the catalase pattern as a screening method. The 4 selected clones contained 100% PEX3 KO cells as estimated by catalase staining. No Pex3 protein was detected by immunoblotting in these clones, and the peroxisome membrane protein PMP70 was also absent, as expected for a protein inserted in peroxisome membrane by a PEX3-dependent mechanism (Hettema et al., 2000). In contrast, the catalase expression levels were normal, in keeping with its cytosolic localization in the absence of peroxisomes (Figure 7A). As clones 1.7 and 1.6C originated from the same PEX3-KO cell line, we only used one of these clones to analyze HCV replication. In the luciferase-based replication assay, no differences in replication were observed between PEX3 KO clones and control Huh-7 cells both for JFH1 (Figure 7B) and DBN3a (Figure 7C). Surprisingly, when infectious titers were measured in the same PEX3-KO clones infected with JFH1, two clones yielded higher titers and the third one a lower titer than control Huh-7 cells at 3 dpi (Figure 7D). These differences are not correlated to Pex3 expression levels and most likely result from clonal differences unrelated to PEX3 gene inactivation. When the same clones were infected with DBN3a, no significant differences from control Huh-7 cells were observed up to 16 dpi, except for the clone 3.2E, which displayed a lower titer than the other two clones and Huh-7 cells at 16 dpi only (Figure 7E). It is worth noting that clone 3.2E is one of the clones that yielded higher titers with JFH1 at 3 dpi (Figure 7D). Again, this difference was attributed to a clonal effect rather than to PEX3 gene inactivation. Altogether, these results confirmed that HCV is able to replicate to near normal level in the absence of peroxisomes.

### 3.7. Impact of HCV infection on peroxisome functions

As peroxisomes do not apparently play any important role in the HCV life cycle, we sought to assess whether HCV infection would have an impact on peroxisome functions. Peroxisomes play a series of unique functions in the cell metabolism, of which we chose to investigate their role in lipid metabolism and in ROS production and degradation, which are two functions potentially related to HCV replication and pathogenesis.

To probe potential peroxisome dysfunction, we assessed whether the lipid metabolism of peroxisomes is altered in cells replicating HCV. The *β*-oxidation of very long chain fatty acids (VLFCA) occurs exclusively in peroxisomes, whereas mitochondria use shorter fatty acids. Therefore, an excess of very long chain fatty acids is indicative of peroxisome dysfunction (Moser et al., 1999). Moreover, the *β*-oxidation of VLCFA is considered the main source of H_2_O_2_ in peroxisomes. To assess peroxisome *β*-oxidation, we measured the total cellular content of C16 and C26 fatty acids in control and in SGR-containing Huh-7 cells, and in PEX3-KO cells as a control of cells with no active peroxisomes. As shown in Supplementary Figure S5, the ratio of C26 fatty acid over C16 fatty acid did not significantly change in cells containing sub-genomic replicons of JFH1 or DBN3a strains, as compared with control Huh-7 cells. In contrast, the C26:C16 ratio was significantly higher in PEX3-KO cells, as expected. These results suggest that the peroxisomal β-oxidation is not significantly affected by HCV infection.

**Figure 8:**
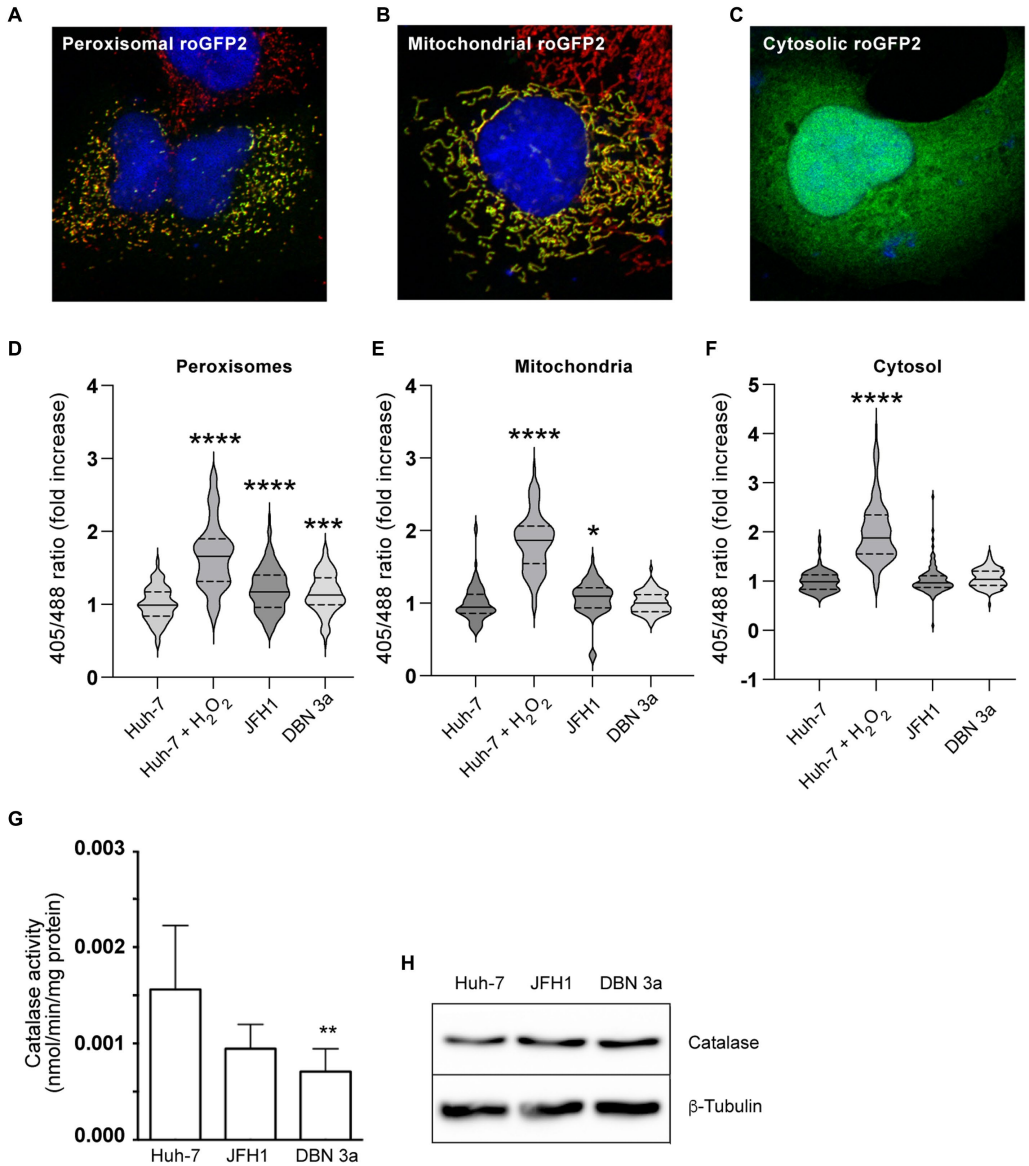
Alteration of ROS metabolism in HCV SGR-containing cells. **(A)** Immunofluorescence of peroxisomal roGFP2 sensor (green) transfected in Huh-7 cells co-stained with PEX14 (red). **(B)** Immunofluorescence of mitochondrial roGFP2 sensor (green) transfected in Huh-7 cells co-stained with TOM20 (red). **(C)** Immunofluorescence of cytosolic roGFP2 sensor (green) transfected in Huh-7 cells. **(D–F)** *In situ* measurements of the oxidation state of roGFP2 sensors in control and HCV SGR-containing Huh-7 cells transiently transfected with a peroxisomal **(D)**, a mitochondrial **(E)** or a cytosolic **(F)** roGFP2-based sensor. Fluorescence images of live cells were acquired and the ratio of fluorescence emitted from 405- and 488-nm excitation were used to calculate the 405/488-nm excitation ratio. At least 90 cells from 3 independent transfections were used for each sample. Ratio values are presented as violin plots with the median and quartiles indicated as solid and dotted lines, respectively. **(G)** Catalase activity in cell lysates of control and SGR-containing Huh-7 cells. **(H)** Immunoblot analysis of catalase and tubulin expression in control and HCV SGR-containing Huh-7 cells. *, *p* < 0.05; **, *p* < 0.01; ***, *p* < 0.001; ****, *p* < 0.0001 (Kruskall-Wallis test).

To specifically survey peroxisomal ROS content, we used a peroxisome-localized roGFP2-based probe (Ivashchenko et al., 2011). For comparison, we used cytosolic and mitochondrial roGFP2 constructs. The 3 probes were expressed in Huh-7 cells and their correct intracellular localizations were verified by immunofluorescence (Figures 8A–C). The roGFP-2 sensor is based on the presence in GFP of 2 surface-exposed cysteine residues at positions 147 and 204. These 2 Cys residues can form a disulfide bond in an oxidative environment, and the presence of this disulfide bond alters the excitation spectrum of the probe (Dooley et al., 2004). Relative to reduced roGFP-2, oxidized roGFP-2 fluorescence excitation is increased at 405 nm and reduced at 488 nm, with no change of emission at 514 nm. Therefore, the ratio of roGFP2 fluorescence signals measured at 405 nm versus 488 nm (ratio 405/488) increases in relation to the oxidative content in the sub-cellular environment containing the probe. When this ratiometric redox sensor was expressed in control and SGR-containing Huh-7 cells, an increased fluorescence ratio was detected with the peroxisomal probe (Figure 8D). A small difference was also observed with the mitochondrial probe for the JFH1-SGR, but not for the DBN3a-SGR (Figure 8E). No difference of fluorescence ratio was observed for the cytosolic probe both for DBN3a-based replicon (Figure 8F). These results indicate that the redox potential of peroxisomes is increased in cells replicating HCV.

To assess whether this increase of ROS in peroxisomes could result from a defect in H_2_O_2_ degradation, we measured the cellular activity of catalase in cell lysates of Huh-7 and SGR-containing cells. As shown in Figure 8G, catalase activity was reduced in cells containing the DBN3a-based replicon as compared with Huh-7 cells, and also appear to be reduced in cells containing the JFH1-based replicon, although the difference was not significant for this strain. This lower activity was not correlated with lower levels of catalase expression in SGR-containing cells, as determined by immunoblotting (Figure 8H). These results suggest that the higher peroxisomal ROS levels in HCV-infected cells could at least in part result from a defect in catalase activity.

## 4. Discussion

Viral infections often result in alterations of the cellular metabolism and of the morphology of intracellular compartments. For positive-strand RNA viruses, which replicate in association with rearranged cytoplasmic membranes, the stress imposed by the establishment of replication complexes can result in an intense reorganization of various intracellular compartments. ER stress and alterations of mitochondria and lipid droplets have previously been documented in HCV-infected cells (Tardif et al., 2004; McLauchlan, 2009; Arciello et al., 2013). In this study, we found that peroxisome morphology and functions are also altered in HCV-infected cells. In line with our results, two recently published multi-omics studies reported peroxisome dysfunctions in cells infected with HCV *in vitro* and in patients samples (Lupberger et al., 2019; Ali et al., 2023).

Using a proximity-based biotinylation approach, we unexpectedly uncovered a close association of peroxisomes and HCV replication complexes coupled to an alteration of peroxisome morphology. Two peroxisome membrane proteins, ACBD5 and PEX14, were detected in the set of proteins biotinylated by HCV NS5A-APEX2 fusion in the context of cells containing a sub-genomic HCV replicon. The biotinylation of these proteins did not represent a relocalization of individual peroxisomal proteins from peroxisomes to replication complexes in infected cells, but rather the recruitment of whole peroxisomes in the vicinity of HCV replication complexes. ACBD5, a protein involved in ER/peroxisome membrane contact sites and in the transfer of lipids between peroxisomes and the ER (Hua et al., 2017; Costello et al., 2017a), ranked second in the list of proteins biotinylated by NS5A-APEX2. This is in keeping with its association to ER proteins VAPA and VAPB, which interact with NS5A in HCV-infected cells. We hypothesized that, for this reason, ACBD5 could be in close proximity to NS5A, through its VAPA/B interaction and that it could be instrumental in the association of peroxisomes and replication complexes. However, no difference of association between these two compartments could be observed in cells lacking ACBD5. This indicates that ACBD5 is either not involved in peroxisome-replication complex association, or that it is part of a larger set of proteins collectively responsible for this association. This second possibility could explain the lack of impact of ACBD5 gene inactivation on peroxisome-HCV replication complex association. Other membrane contact sites, such as ER-plasma membrane or ER-mitochondria membrane contact sites are known to involve numerous proteins (Wu et al., 2018). Not much is known about the protein composition of ER-peroxisome membrane contact sites in human cells, except that ACBD5 (Hua et al., 2017; Costello et al., 2017a) and a peroxisomal ACBD4 isoform (Costello et al., 2017b) are involved.

In addition to peroxisomes recruitment to the vicinity of HCV replication complexes, an alteration of peroxisome morphology was also observed both in HCV-infected and SGR-containing cells. These morphological alterations included enlarged peroxisomes and also extended tubules. Tubules were mostly visible in SGR-containing cells, whereas enlarged peroxisomes were present in both conditions. The appearance of extended tubules in SGR-containing cells but not very frequently in infected cells could result from a longer time of HCV replication in SGR-containing cells, as compared to infected cells, and could therefore represent a more advanced phenotype than the one visible in infected cells, which were kept in culture for a maximum of 16 days in our experiments. Alternatively, the difference of phenotype could also result from the difference of intracellular localization of HCV replication complexes in infected cells, in which they are recruited to lipid droplet-associated virion assembly sites via NS5A interaction with core (Miyanari et al., 2007). In contrast, replication complexes are not associated to lipid droplets in SGR-containing cells. The presence of peroxisomes with an altered morphology did not completely correlate with their association with replication complexes, suggesting that their recruitment is not likely to be the only trigger of their change of morphology. The metabolic state of cells can influence peroxisome size and number (Farré et al., 2019). HCV infection increases glucose metabolism and the STAT3 signaling pathway and it has been suggested that these metabolic changes reduce peroxisome function (Lupberger et al., 2019). We could hypothesize that these metabolic changes could also impact peroxisome morphology.

The turnover of peroxisomes is quite short (Islinger et al., 2018; Farré et al., 2019). Peroxisomes are selectively degraded by a special mode of autophagy named pexophagy, and new peroxisomes are mostly generated by growth and fission. An alternative mode of *de novo* production of peroxisomes also exists by fusion of two pre-peroxisomal vesicles originating from ER and mitochondria (Sugiura et al., 2017). Our results indicate a reduction in the number of peroxisomes in infected cells, as previously reported (Lupberger et al., 2019). However, we also observed that the mean volume of peroxisomes is increased in HCV-infected cells and the total volume occupied by peroxisomes per cell does not appear to significantly change overtime during infection. This suggests that the change of peroxisome morphology is not likely to result from increased or decreased peroxisome turnover in HCV-infected cells. A defect in peroxisome fission could result in a phenotype similar to that observed in infected cells. The presence of elongated tubule-like peroxisomal structures in SGR-containing cells also points to a possible defect in peroxisome fission. In yeast, the deletion of genes involved in peroxisome fission results in an increased size and a reduced number of peroxisomes (Yofe et al., 2017). Alternatively, larger structures could result from peroxisome clustering and aggregation. It would be interesting to image peroxisome alterations by electron microscopy. However, peroxisomes are small single membrane-bound organelles ranging in size from 0.1 to 1 μm, that are not easy to identify in electron microscopy in human cells, in contrast to rodent peroxisomes, easily identifiable by the presence of a prominent crystalloid core, which is absent in human cells.

Peroxisomes are not essential for cell viability, which made it possible to evaluate their implication in the establishment of HCV infection. Surprisingly, no impact on the HCV life cycle was observed in cells devoid of peroxisomes for the two strains we used up to 16 dpi, despite the alterations of their morphology and of peroxisomal ROS metabolism during the same period in normal peroxisome-containing cells. Other viruses have been reported to interact with peroxisomes. Rotavirus VP4 protein contains a PST1 motif and is localized in peroxisomes (Mohan et al., 2002), but the importance of peroxisomes in rotavirus infection remains undetermined. Viruses of the *Herpesviridae* family have been shown to increase peroxisome numbers in infected cells (Sychev et al., 2017; Jean Beltran et al., 2018). Interestingly, for human cytomegalovirus, it has been shown that the biogenesis of peroxisomes by fission is enhanced and coupled to alterations in peroxisome morphology, protein composition and lipid metabolism, which result in an increased production of plasmalogens that are required for virion assembly (Jean Beltran et al., 2018). This increase in plasmalogens production is linked to a reorganization of ER/peroxisome contact sites and an increased association of peroxisomes to the ER membrane (Cook et al., 2022). Other viruses, such as Dengue virus and West Nile virus, two viruses of the *Flaviviridae* family, cause a decrease in peroxisome number by targeting their capsid protein to the peroxisomal membrane (You et al., 2015), a mechanism different from that of HCV, for which the core protein, which is absent from SGRs, does not appear to be involved in peroxisome alteration. This decrease in peroxisomes in flavivirus-infected cells results in an inhibition of type III interferon expression. Indeed, it has been shown that the innate immunity adaptor MAVS is located both on mitochondrial and peroxisomal membranes and promotes different antiviral responses at each location (Dixit et al., 2010). Moreover, mitochondrial MAVS preferentially induces a type I interferon response, whereas peroxisomal MAVS exclusively induces a type III interferon response (Odendall et al., 2014). HCV uses another mechanism to counteract MAVS signaling. In HCV-infected cells, MAVS is cleaved by NS3 both on mitochondrial and peroxisomal membranes (Bender et al., 2015), and this cleavage inhibits all interferon responses (Foy et al., 2005; Meylan et al., 2005).

In HCV-infected cells, we did not detect any alteration of the lipid metabolism part, which deals with the β-oxidation of VLCFA, a metabolic pathway exclusively performed by peroxisomes. This finding is at odds with lipidomics studies of patient samples, in which an excess of VLCFAs was reported (Lupberger et al., 2019; Ali et al., 2023). The reason for this discrepancy is still unclear, but may have to do with the different system used (infectious virus and SGR). It would be interesting to investigate if any of the HCV proteins absent in SGR could impact peroxisomal β-oxidation, either by itself or in combination with proteins of the HCV replication complex. In contrast, we found an alteration of peroxisomal redox potential during HCV infection. Our results indicate that ROS levels are specifically increased in peroxisomes. This result suggests that peroxisomes are the major site of ROS increased production in HCV SGR-containing cells. HCV core has been reported to induce the production of ROS (Okuda et al., 2002) through mitochondrial electron transport inhibition (Korenaga et al., 2005; T. Wang et al., 2010). This has led to the idea that mitochondria are the major site of ROS production in HCV-infected cells. The use of SGRs, which do not express core, allowed us to uncover the additional role of non-structural proteins in ROS production by peroxisomes. The β-oxidation of VLCFA by peroxisomes is a source of H_2_O_2_ production in peroxisomes. However, our results do not indicate that VLCFA accumulate or are more actively degraded in HCV SGR-containing cells. Rather, a lower rate of ROS elimination by catalase (and possibly other detoxifying enzymes) appears to be a possible cause of ROS accumulation in peroxisomes, and this loss of catalase activity is not related to a decreased catalase expression. It would be interesting to decipher the mechanism of catalase inhibition and its impact on ROS accumulation and pathogenesis induced. Interestingly, peroxisomes are altered and catalase activity is reduced in hepatocellular tumors (Litwin et al., 1999). Given the role of oxidative stress in cancer development and progression (Cheung and Vousden, 2022), peroxisomes may have an as yet underestimated contribution to these processes.

In conclusion, our study indicates that peroxisome function and morphology are altered in HCV-infected cells. These perturbations are likely to contribute to liver dysfunction and pathogenesis in chronically infected patients.

## Data availability statement

The original contributions presented in the study are included in the article/Supplementary material, further inquiries can be directed to the corresponding author.

## Author contributions

EM: Conceptualization, Investigation, Methodology, Writing – review & editing. NC: Investigation, Methodology, Writing – review & editing. J-MS: Investigation, Writing – review & editing. MF: Writing – review & editing, Formal analysis. OD: Writing – review & editing, Investigation. NB: Investigation, Writing – review & editing. QT: Investigation, Writing – review & editing. SR: Writing – review & editing, Resources. JB: Resources, Writing – review & editing. LC: Writing – review & editing. JB-M: Writing – review & editing, Investigation. GM: Writing – review & editing, Data curation. YS: Conceptualization, Funding acquisition, Investigation, Methodology, Writing – review & editing. JD: Conceptualization, Writing – review & editing. YR: Conceptualization, Funding acquisition, Investigation, Methodology, Writing – original draft.

## Funding

This work was supported in part by grants from the CPER-CTRL (Centre Transdisciplinaire de Recherche sur la Longévité) program (to YR and YS) and from the French Agence Nationale de Recherche sur le Sida et les Hépatites Virales (ANRS) (to YR). EM was supported by a pre-doctoral fellowship from ANRS.

## Conflict of interest

The authors declare that the research was conducted in the absence of any commercial or financial relationships that could be construed as a potential conflict of interest.

## Publisher’s note

All claims expressed in this article are solely those of the authors and do not necessarily represent those of their affiliated organizations, or those of the publisher, the editors and the reviewers. Any product that may be evaluated in this article, or claim that may be made by its manufacturer, is not guaranteed or endorsed by the publisher.

## Footnotes

^1^ http://proline.profiproteomics.fr
