# Supplementary figures and images for: Hepatitis C virus alters the morphology and function of peroxisomes
Source: Front Microbiol. 2023 Sep 21;14:1254728. doi: 10.3389/fmicb.2023.1254728 (PMC10551450; doi:10.3389/fmicb.2023.1254728)

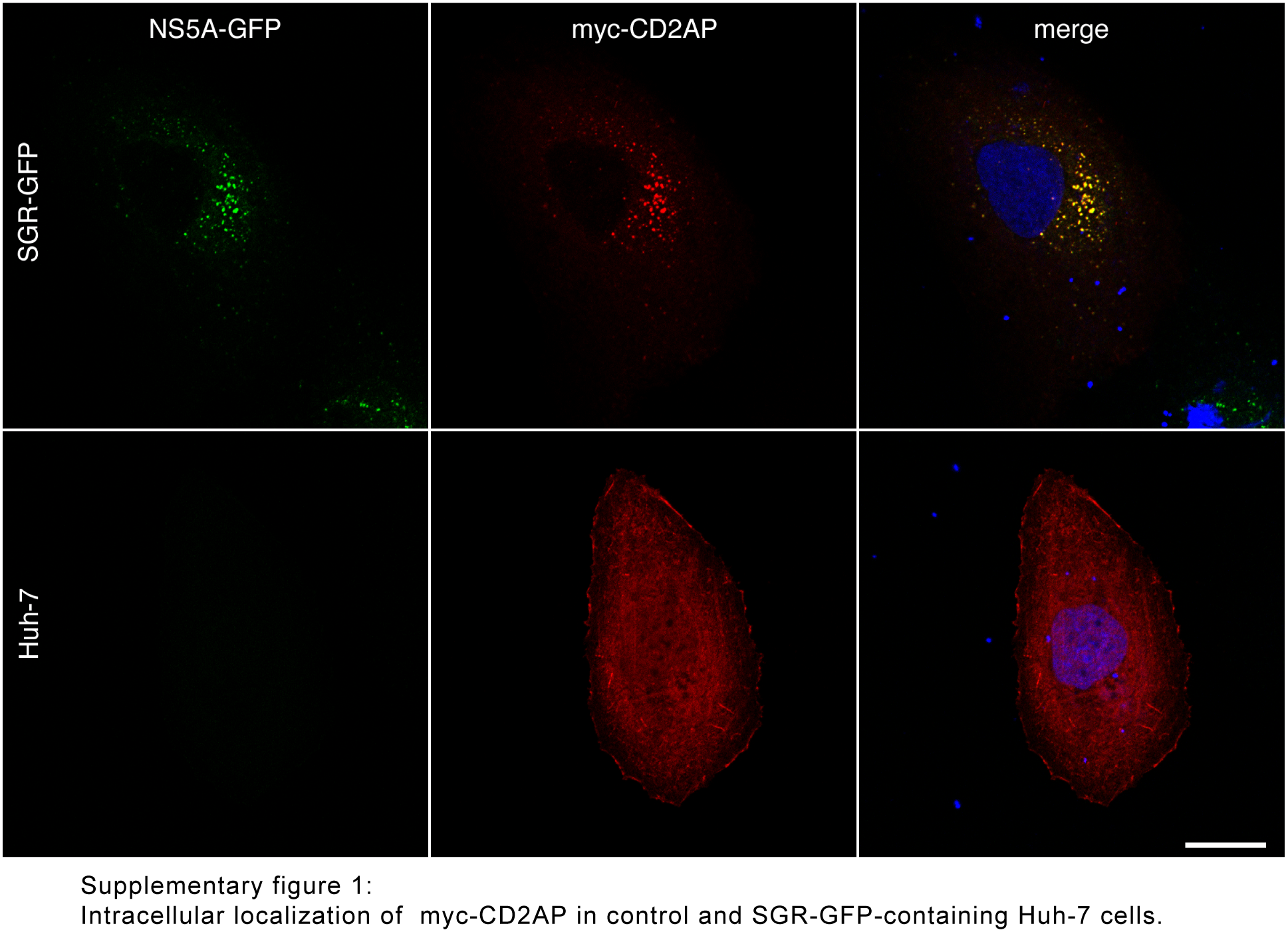

Supplement: Supplementary file 1 [file Image_1.TIF]

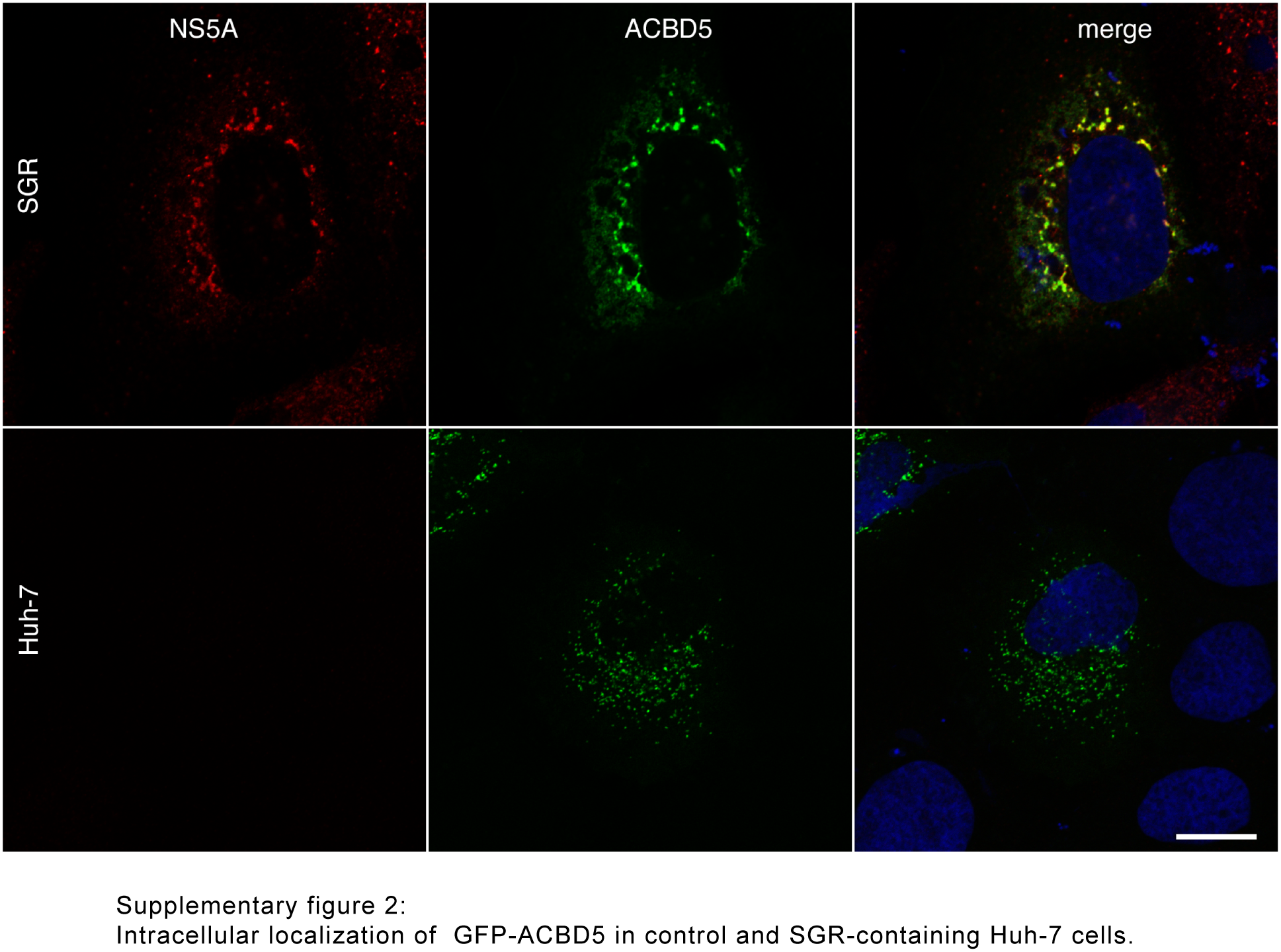

Supplement: Supplementary file 2 [file Image_2.TIF]

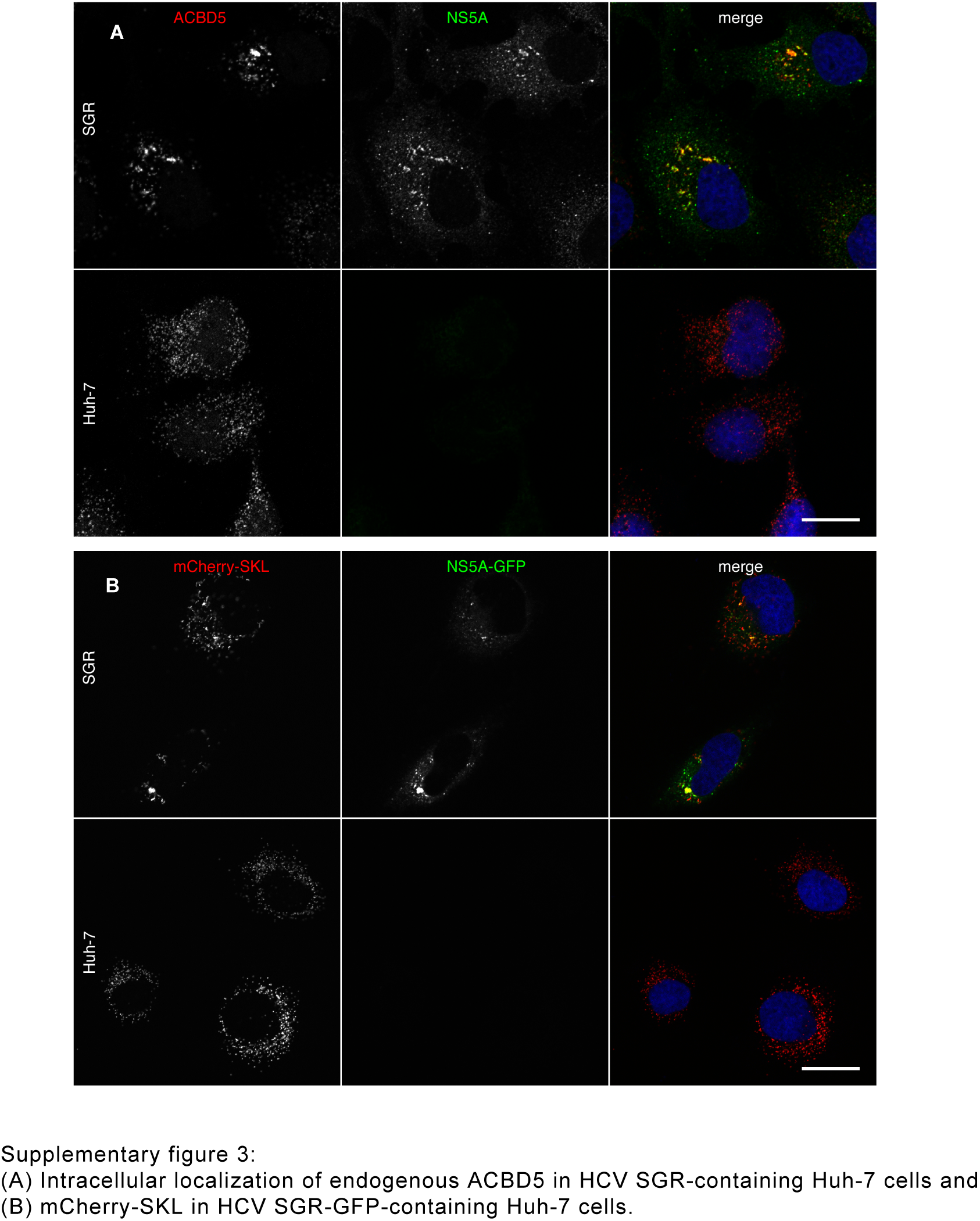

Supplement: Supplementary file 3 [file Image_3.TIF]

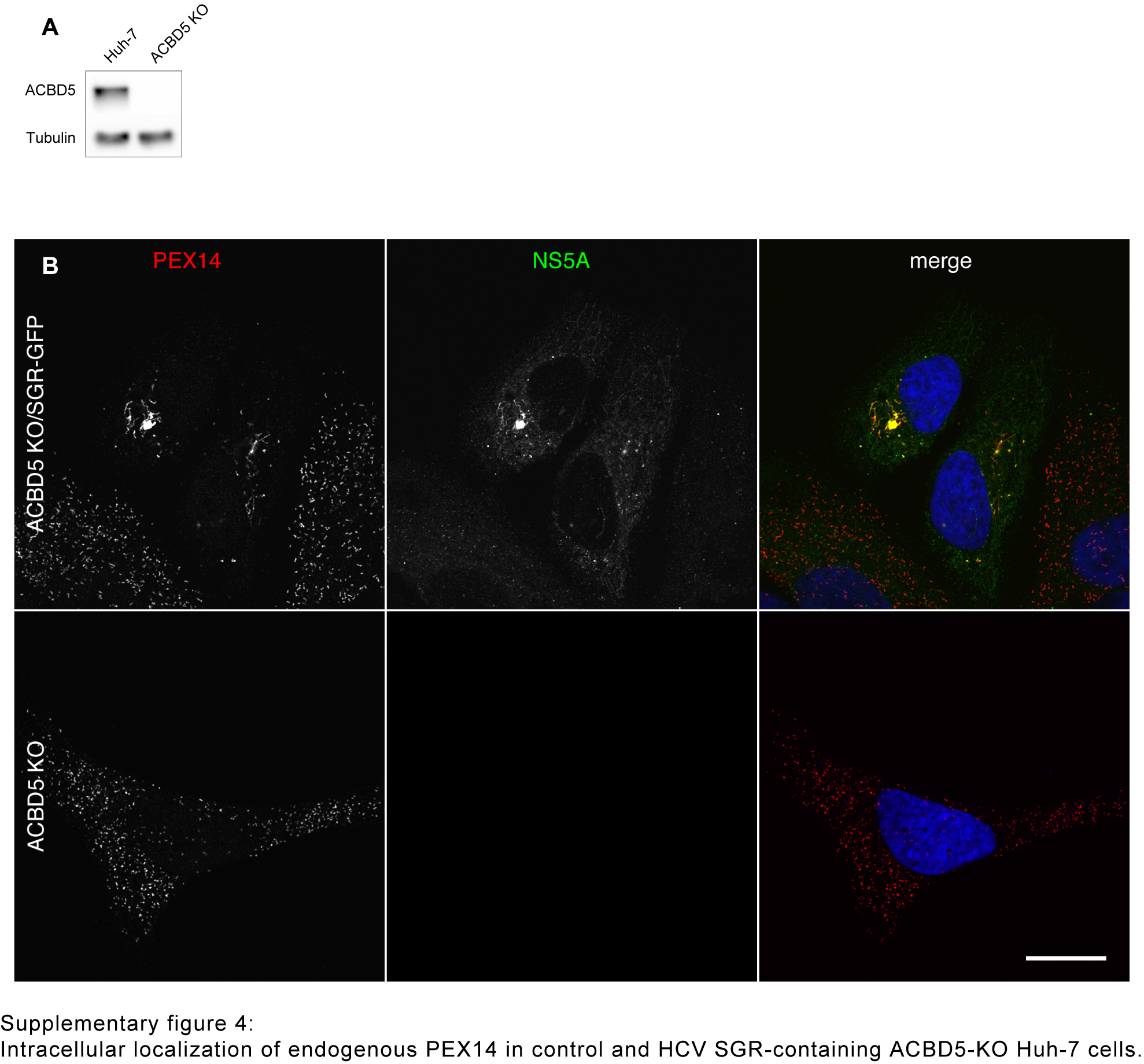

Supplement: Supplementary file 4 [file Image_4.TIF]

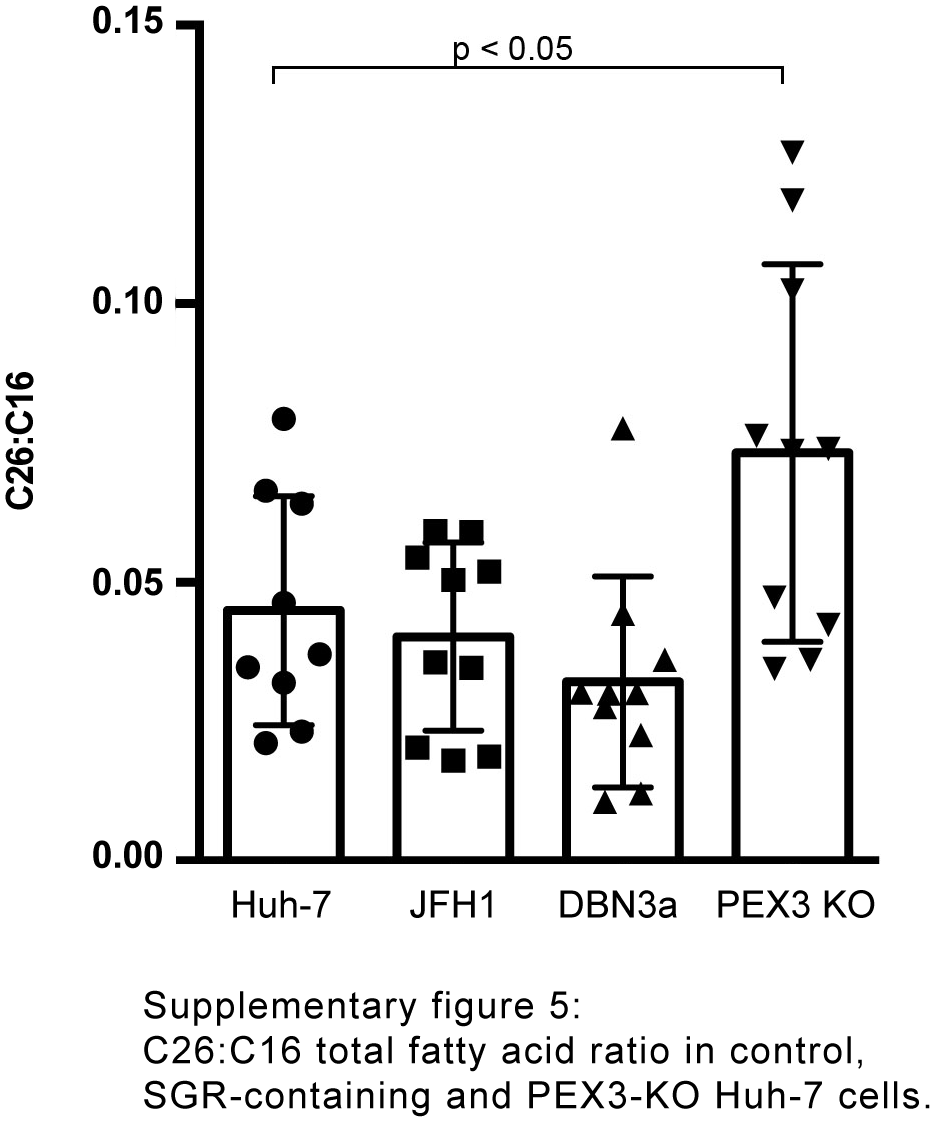

Supplement: Supplementary file 5 [file Image_5.TIF]
